# Comparative transcriptomics of a generalist aphid, *Myzus persicae* and a specialist aphid, *Lipaphis erysimi* reveals molecular signatures associated with diversity of their feeding behaviour and other attributes

**DOI:** 10.3389/fpls.2024.1415628

**Published:** 2024-12-02

**Authors:** Manvi Sharma, Praveen Kumar Oraon, Rakesh Srivastava, Rubina Chongtham, Shailendra Goel, Manu Agarwal, Arun Jagannath

**Affiliations:** Department of Botany, University of Delhi, Delhi, India

**Keywords:** aphids, generalist, specialist, transcriptome, differential expression, effectors

## Abstract

**Introduction:**

Aphids are phloem sap-sucking insects and are a serious destructive pest of several crop plants. Aphids are categorized as “generalists” or “specialists” depending on their host range. *Myzus persicae* (Sulz.) is a generalist aphid with a broad host range while *Lipaphis erysimi* (Kalt.), a specialist aphid, has a narrow host range. Aphid infestation involves several sequential stages including host recognition and selection, overcoming primary plant defence barriers, feeding on phloem sap and detoxification of host defence responses. Information on the molecular basis of variations between generalist and specialist aphids with reference to the above processes is limited.

**Methods:**

In the current study, we generated transcriptome data of *M. persicae* and *L. erysimi* from adult and nymph stages and analysed the differential expression of genes between adults of the generalist and specialist aphid and similarly, between nymphs of the two aphid species. We categorized these differentially expressed genes into nine different categories namely, chemosensation-related, plant cell wall degrading enzymes, detoxification-related, digestive enzymes, peptidases, carbohydrate-, lipid-, amino acid-metabolism and reproduction. We also identified putative effector molecules in both *M. persicae* and *L. erysimi* from the transcriptome data,

**Results and discussion:**

Gene expression analysis identified 7688 and 8194 differentially expressed unigenes at adult and nymph stages, respectively of *M. persicae* and *L. erysimi. M. persicae* showed significantly higher levels of expression in a greater number of unigenes (5112 in adults and 5880 in nymphs) in contrast to the specialist, *L. erysimi* (2576 in adults and 2314 in nymphs) in both developmental stages. In addition, *M. persicae* displayed a greater number (350 in adults and 331 in nymphs) of upregulated unigenes involved in important processes such as host recognition, plant cell wall degradation, detoxification, digestion and metabolism, which correlate with its dynamic and polyphagous nature in contrast to the specialist (337 in adults and 251 in nymphs). We also observed a greater number of putative effectors in *M. persicae* (948 in adults and 283 in nymphs) than *L. erysimi* (797 in adults and 245 in nymphs). Based on our analysis, we conclude that the generalist aphid, *M. persicae* has a more diversified and stronger arsenal of genes that influence its polyphagous feeding behaviour and effective response to plant defence mechanisms against insect-herbivory. Our study provides a compendium of such candidate genes that would be most useful in studies on aphid biology, evolution and control.

## Introduction

Aphids are small, soft-bodied, phloem sap-sucking insects belonging to the Order Hemiptera. They consist of approximately 4000 species which collectively infect ~25% of plant species worldwide (Dixon, 1985). Of these, around 100 aphid species infect a wide range of crop plants including oilseeds, cereals, pulses, vegetables and fruits leading to significant losses in crop productivity and yield (Blackman and Eastop, 2007). They also function as vectors for transmission of plant viruses with ~50% of insect-borne plant viruses being transmitted by aphids (Katis et al., 2007). Aphids can multiply by sexual reproduction but more importantly, they also have the ability to reproduce parthenogenetically which results in rapid colonisation, multiplication and spread rendering them highly destructive to standing crops. Parthenogenetic females give rise to young nymphs which develop into adults through four instar stages. Aphids are classified as generalists or specialists based on their host range. Specialists exhibit a narrow and selective host range while generalists are polyphagous and capable of infesting a wide diversity of plant species allowing them access to a wider range of resources (Capinera, 2008). Around 99% of all aphid species have a high degree of host specificity and the remaining 1% are highly polyphagous (Egas et al., 2005).

Infestation by aphids begins with detection of a suitable host using their chemosensory system, which includes various protein/gene families such as odorant binding proteins, odorant binding receptors, chemosensory proteins and gustatory receptors (Zhang et al., 2020). Following identification of a potentially favourable host, aphids initiate the feeding process by inserting a highly specialised stylet into the host tissue through which, they release a watery saliva. Aphids overcome the first line of host defence i.e., the plant cell walls by disrupting cell wall polymers using plant cell-wall degrading enzymes (PCWDEs) present in the saliva (Guangxi et al., 2006; Thorpe et al., 2016; Silva-Sanzana et al., 2020). A gelling saliva is also secreted around the stylet to facilitate penetration and to minimise physiological contact with host cells (Tjallingii, 2006). The penetrating stylet also punctures adjacent cells during its progression towards the phloem to analyse the host cell sap for its pH, sugar concentration and amino acid contents to determine suitability of the host plant for feeding. The watery saliva contains salivary effectors that are proteins or small molecules which modify host cell physiology and can induce and/or suppress defence responses in the host plant (Hogenhout et al., 2009; Mondal, 2017, 2020). Several studies have identified effectors in various aphids viz., *Myzus persicae* (Harmel et al., 2008), *Acyrthosiphon pisum* (Carolan et al., 2011), *Schizaphis graminum* (Cooper et al., 2011), *Sitovian avenae* (Rao et al., 2013), *Diuraphis noxia* (Nicolis et al., 2022), *Pseudoregma bambucicola* (Zhang et al., 2023) and some of these have been functionally characterised. The phloem sap contains secondary metabolites and other proteins viz., redox regulators, phytohormones-related proteins, protease inhibitors, lectins, etc. that are involved in plant defence (Kehr, 2006). Aphids combat the effects of such proteins using detoxification enzymes, digestive enzymes, and peroxidases (Ramsey et al., 2010). Aphids also show physiological adaptations to feed continuously on a sugar-rich and nitrogen-deficient phloem sap that contains only a few free amino acids, mainly, asparagine, serine, glutamate, aspartate and glutamine as the nitrogen source (Douglas, 2006). They produce proteolytic enzymes to allow digestion of ingested proteins (Pyati et al., 2011).

Several studies based on transcriptome profiling of aphids have attempted to analyse the molecular basis of aphid infestation, growth and development. Transcriptomic studies of bamboo aphid by Zhang et al. (2023) identified putative effectors important for plant-aphid interactions such as those involved in detoxification, digestion and antioxidant enzymes. Transcriptome profiles of alimentary canal of *Sitobion avenae* at pre- and post- feeding stages identified five novel candidate genes that led to higher aphid mortality and delayed development when used as targets in plant-mediated RNAi for aphid control in wheat (Zhang et al., 2013). To study the differences in two generalists feeding on the same host plant, transcriptome studies were conducted by Koch et al. (2019) between *S. graminum* and *Sipha flava* in which, one of the generalists, *S. graminum* upregulated more stress-responsive genes as compared to the other when fed on the same variety of switchgrass. In another study by Wang et al. (2014), comparison of the transcriptome of *S. avenae* (a specialist) and mRNA sequences of *A. pisum* predicted positive selection of 186 pairs of orthologous groups involved in xenobiotics and secondary metabolism which might be responsible for their divergence. In an integrative study combining transcriptomics and proteomics approaches on three different aphid species, *M. persicae*, *M. cerasi* and *Rhopalosiphum padi*, core effectors comprising of previously identified candidate effectors and species-specific effectors were identified (Thorpe et al., 2016).

To the best of our knowledge, comparative transcriptome profiling between generalist and specialist aphids feeding on the same host plant has not been reported till date. Additionally, information on differential expression profiles during infestation in feeding adults and nymphs of generalist and specialist aphids is limited. Comparison between the repertoire of effectors and their expression profiles between a generalist and a specialist aphid has also not been reported. In the current study, we generated and analysed transcriptome data at both adult and nymph stages of development for a generalist, *M. persicae* and a specialist, *Lipaphis erysimi* both of which are devastating pests of the important oilseed crop, *Brassica juncea* (Indian mustard). *L. erysimi* (mustard aphid) specifically feeds on Brassica sp. while *M. persicae* infests host plants of over 50 families encompassing more than 400 species (Weber, 1985; Blackman and Eastop, 2007). These insects also demonstrate significant differences in their infestation behaviour on the same susceptible host (*B. juncea*) during the crop growing season (October – March). In contrast to infestation by the specialist, infestation by the generalist is more regular in successive growing seasons, more widespread (more number of plants are infested with *M. persicae*) and higher numbers of these aphids are found on infested plants. The specialist (*L. erysimi*) was more susceptible to temperature variations and the duration of infestation was lesser compared to the generalist which was able to tolerate fluctuating weather conditions and survive on the host for a longer duration. To understand the basis of these differences in insect behaviour and to decipher the molecular basis of variations in their host range, feeding behaviour, detoxification mechanisms and evolutionary divergence in important genes, we analysed the differential expression of genes between the two aphid species in both adults and nymphs. We also identified putative effector proteins using a bioinformatic pipeline to study differences between the two types of aphids, their role in countering plant defence compounds and the degree of divergence between them. Our study has identified several species-specific novel effectors and generated resources for both the aphids at two different developmental stages which provide interesting leads on aphid biology, divergence and evolution.

## Materials and methods

### Aphid rearing and collection

Apterous adults of *L. erysimi* and *M. persicae* were collected from *Brassica juncea* var. Varuna plants during the growing season and placed inside clip-cages attached to the leaves. After 10 days, offspring thus produced by the parthenogenetic apterous females were picked with a fine brush and transferred to a new clip cage to generate a single clonal lineage. Nymphal (N1 to N4) stages were pooled together while apterous female adults were harvested separately. Aphids were collected directly from clip cages, immediately flash-frozen in liquid nitrogen, and stored at -80°C for RNA extraction. Three biological replicates of both the stages i.e., adult and nymph were harvested.

### Total RNA extraction and sequencing

Total RNA was extracted from ~50 morphs in each sample using RNeasy^®^ Mini Kit (Qiagen, Hilden, Germany) following manufacturer’s instructions. Total extracted RNA was quantified using Qubit 4.0 fluorometer (Thermo Fisher Scientific, Waltham, MA, USA) and its integrity and quality were assessed using Tapestation 2200 (Agilent, CA, USA). Purified total RNA was used for transcriptome library preparation using NEBNext^®^ Ultra™ II Directional RNA Library Prep Kit for Illumina^®^ (New England Biolabs, Ipswich, MA, USA). cDNA library was quantified using Qubit 4.0 fluorometer (Thermo Fisher Scientific, Waltham, MA, USA) and quality assessment was done by Tapestation 2200 (Agilent Technologies, Santa Clara, CA, USA). Transcriptome sequencing was performed on Illumina NovaSeq 6000 platform (Illumina, San Diego, CA, USA) with 151-base pair (bp) paired-end reads.

### Data assembly and annotation

Raw reads were processed to filter low-quality reads and adaptor sequences using Fastp (v0.23.2.0) (Chen et al., 2018) with a phred score cut-off of 30 and a minimum length of 75 bp. High-quality reads thus retained of all the three replicates of adults and nymphs of both the aphid species were catenated together (forward R1, reverse R2 separately) and were assembled *de novo* using Trinity assembler (v2.11.0) (Grabherr et al., 2011) for adult and nymph samples of *L. erysimi*. For adult and nymph samples of *M. persicae*, the reads were first aligned to publicly available reference genome of *M. persicae* MPER_G0061.0 (GCF_001856785.1). Aligned reads were assembled using Trinity assembler in –genome_guided_bam mode. Final transcriptome assembly was generated after performing clustering using CD-HITest (v4.8.1) (Li and Godzik, 2006) at 80% similarity. The completeness of the transcriptome assembly was assessed using BUSCO (Benchmarking Universal Single-Copy Orthologs) (v5.4.7) (Simão et al., 2015) against insecta_odb10 database. Assembled unigenes were functionally annotated using the Blast2GO program against public databases NCBI non-redundant (Nr) and AphidBase (‘local database’ was created by combining Nr and aphidbase data against which blast was performed). Functional classification of all the unigenes including Gene Ontology (GO) was perform using Blast2GO. Kyoto Encyclopedia of Genes and Genomes (KEGG) pathway analysis was performed using DAVID (Sherman et al., 2007).

### Identification of differentially expressed genes

Differentially expressed transcripts between adults and nymphs (adult vs nymph) of both the species as well as between the same stages of the two species (adult vs adult; nymph vs nymph) were identified using R- based tool DESEQ2 (v1.36.0.5) (Love et al., 2014) by comparing normalised Fragments Per Kilobase of transcripts per Million mapped reads (FPKM) count generated by RSEM (Li and Dewey, 2011). Transcripts that showed expression levels in at least two of the replicates were selected for differential gene analysis. The resulting p-values were adjusted to control the false discovery rate using Benjamini-Hochberg method. Among the adults or nymphs of two species, transcripts with log2fold change >=2 or <2 P_adjusted_- value less than 0.05 were considered as upregulated or downregulated respectively among differentially expressed genes (DEGs).

### *In silico* identification of effectors

To predict putative effector molecules from transcriptome data of *L. erysimi* and *M. persicae*, we used the pipeline described by Prajapati et al. (2020) and Min (2010). All the assembled transcripts were subjected to clustering using CDHIT-est to remove any redundancy. These unigenes were analysed using TransDecoder (https://github.com/TransDecoder/TransDecoder.git) for ORF calling with a cut off value of minimum 100 amino acids and all the possible ORFs were translated to proteins. Predicted proteins thus obtained were used for the identification of candidate effector molecules in both the aphid species. The predicted proteins were filtered for the presence of signal peptide using SignalP (Petersen et al., 2011) (SignalP package in InterProScan). Filtered proteins were further analysed for the presence of transmembrane domain using InterProScan (v5.62.94) (Jones et al., 2014) (TMHMM package in InterProScan). Finally, subcellular localisation was predicted by TargetP-2.0 (Emanuelsson et al., 2000) and WolfPSort (Horton et al., 2007). Protein sequences which showed the presence of signal peptide in their sequence, absence of transmembrane domain and predicted to be extracellular (ext >17, Prajapati et al., 2020) using WolfPSort were considered putative effector proteins.

### Orthology and Ka/Ks analysis

The coding sequence (CDS) of each unigene was determined using TransDecoder and Blastx was conducted (with cutoff E-value of 1e-5, 50% of query coverage) to compare the CDS of unigenes against predicted genes of pea aphid. Single copy orthologous genes between *L. erysimi* and *M. persicae* were identified using OrthoFinder (v2.5.5) (Emms and Kelly, 2019). The CDS region of predicted single copy orthologs were aligned by MAFFT (v7.505) (Katoh and Standley, 2013) using – auto parameter which automatically ran on L-INS-i mode and the alignments were trimmed using trimAl (v1.4.rev15) (Capella-Gutiérrez et al., 2009) with “gappyout” option to remove poorly aligned sequences. The substitution rates between these single copy orthologous pairs were determined separately for non-synonymous sites i.e. Ka and synonymous sites i.e. Ks using approximate method in KaKs_Calculator3 (Zhang, 2022). YN method was used to perform pairwise approximate method. Since the sequencing errors were distributed equally among synonymous and non-synonymous sites, they were not expected to influence the results in this analyses (Tiffin and Hahn, 2002).

### Real-time quantitative PCR

To conduct qRT-PCR analysis, total RNA was extracted from two biological replicates of four aphid samples (*M. persicae* adult, *L. erysimi* adult, *M. persicae* nymph, *L. erysimi* nymph) and its integrity was checked using agarose gel electrophoresis after performing DNase I (Thermo Fischer Scientific, Inc., Waltham, MA, USA) treatment. First-strand cDNA synthesis was performed from 2μg of purified RNA using iScript™ cDNA synthesis kit (Bio-Rad, Hercules, CA, US) following manufacturer’s protocol. Primer sequences for transcripts to be validated were designed using IDT OligoAnalyzer™ Tool (Integrated DNA Technologies, Newark, New Jersey, US) software and are provided in **Supplementary File 3**. The *40S ribosomal protein S23* gene was used as endogenous gene control (primer sequence provided in **Supplementary File 3**) to stabilise expression levels among all the replicates of aphid samples (Yang et al., 2015), each with two technical replicates. After normalisation with endogenous control gene, the relative expression levels of target transcripts were analysed by ΔΔCt method by pairwise comparison between *M. persicae* adult with *L. erysimi* adult and *M. persicae* nymph with *L. erysimi* nymph. qRT-PCR was performed on CFX Connect Real-Time System (Bio-Rad, Hercules, CA, US). To determine the statistical significance of qRT-PCR data, *t-test* was conducted (p_value_ < 0.05).

## Results and discussion

### Illumina sequencing, transcriptome assembly and functional annotation

We generated transcriptome data of *L. erysimi* and *M. persicae* from two stages of aphid development - adults and nymphs. We obtained an average of 37.16, 28.53, 29.48 and 32.67 million raw reads of *L. erysimi* adult, *M. persicae* adult, *L. erysimi* nymph and *M. persicae* nymph, respectively. The raw reads were processed for quality screening. The clean, filtered reads thus obtained were used for assembly and further clustering of transcripts that resulted in unigenes with an average length of 1096.94 bp, 1345.54 bp, 1440.63 bp, 1532.69 bp for *L. erysimi* adult, *M. persicae* adult, *L. erysimi* nymph and *M. persicae* nymph, respectively (**Table 1**). High BUSCO scores for all samples indicated a high degree of completeness and quality of the assembly. BUSCO scores were comparable for *M. persicae* and *L. erysimi* adults (90.9% and 90.5%, respectively) and nymphs (85% and 86.3%, respectively) (**Table 1**). N50 values were also greater than 2 kb indicating the superior quality of all the assemblies.

**Table 1:** Summary of transcriptomic data generated in *Myzus persicae* and *Lipaphis erysimi* at adult and nymph stage.

	*M. persicae* adult	*M. persicae* nymph	*L. erysimi* adult	*L. erysimi* nymph
Average Raw reads (bp)	28533626	32677230	37166324	29486830
Average Filtered read (bp)	27338676	31670028	33655556	28691401
Total unigenes	27420	29642	42963	33656
Total assembled bases	36894745	45431928	47127835	48485979
Average sequence length (bp)	1345.54	1532.69	1096.94	1440.63
N50 value (bp)	2367	2763	2126	2634
GC%	35.52	33.59	34.97	33.98
Complete BUSCOs	90.9%	85.0%	90.5%	86.3%
Complete and single copy BUSCOs	75.3%	68.7%	64.8%	61.0%
Complete and duplicated BUSCOs	15.6%	16.3%	25.7%	25.3%
Fragmented BUSCOs	1.0%	1.5%	4.0%	2.0%
Missing BUSCOs	8.1%	13.5%	5.5%	11.7%

**Figure 1:**
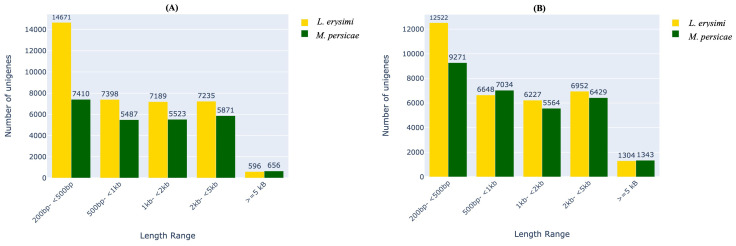
Distribution of unigenes based on their lengths in *L. erysimi* and *M. persicae* **(A)** adults and **(B)** nymphs. Numerals above the bars indicate the number of genes in that category in each aphid species.

Analysis of unigenes in different categories of transcript lengths indicated that *L. erysimi* had a larger number of unigenes in most categories as compared to *M. persicae* in both adults and nymphs (**Figure 1**). This could be attributed to *de novo* assembly of *L. erysimi* transcriptome in contrast to the *M. persicae* transcriptome for which, reference-based assembly was done. These results are in consonance with an earlier study on aphid genomes (Mathers et al., 2017) which showed higher number of genes in *Acyrthosiphon pisum*, a specialist aphid, due to extensive duplication of genes from conserved gene families and four-times the number of lineage-specific genes than in *M. persicae*, a generalist. However, for transcripts >=5kb, we observed more unigenes in both adults and nymphs of the generalist, *M. persicae* than *L. erysimi*. Among transcripts in the 500bp -1kb length range, more unigenes were found in nymphs of *M. persicae* as compared to *L. erysimi* (**Figure 1**). Unigenes were annotated against multiple databases including merged NCBI-Nr and aphidbase, GO, KEGG, PFAM and EC number databases. Approximately 64.7%, 64.2%, 61.8% and 53.1% of total unigenes of *L. erysimi* adult, *M. persicae* adult, *L. erysimi* nymph and *M. persicae* nymph, respectively were annotated by at least one of the above databases (**Supplementary Table 1**). Many unigenes were annotated with multiple databases.

**Figure 2:**
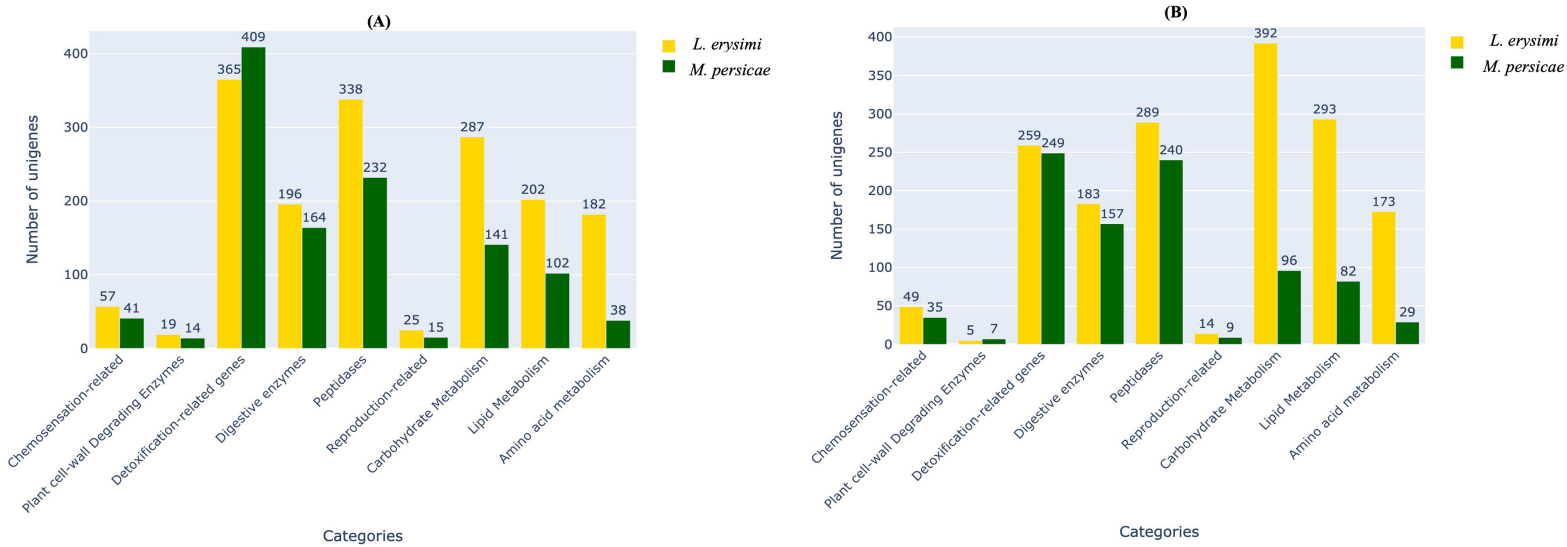
Distribution of unigenes under important functional categories of aphid-plant interactions in *L. erysimi* and *M. persicae* at **(A)** adult and **(B)** nymph stages. Numerals above the bars indicate the number of genes in that category in each aphid species.

**Figure 3:**
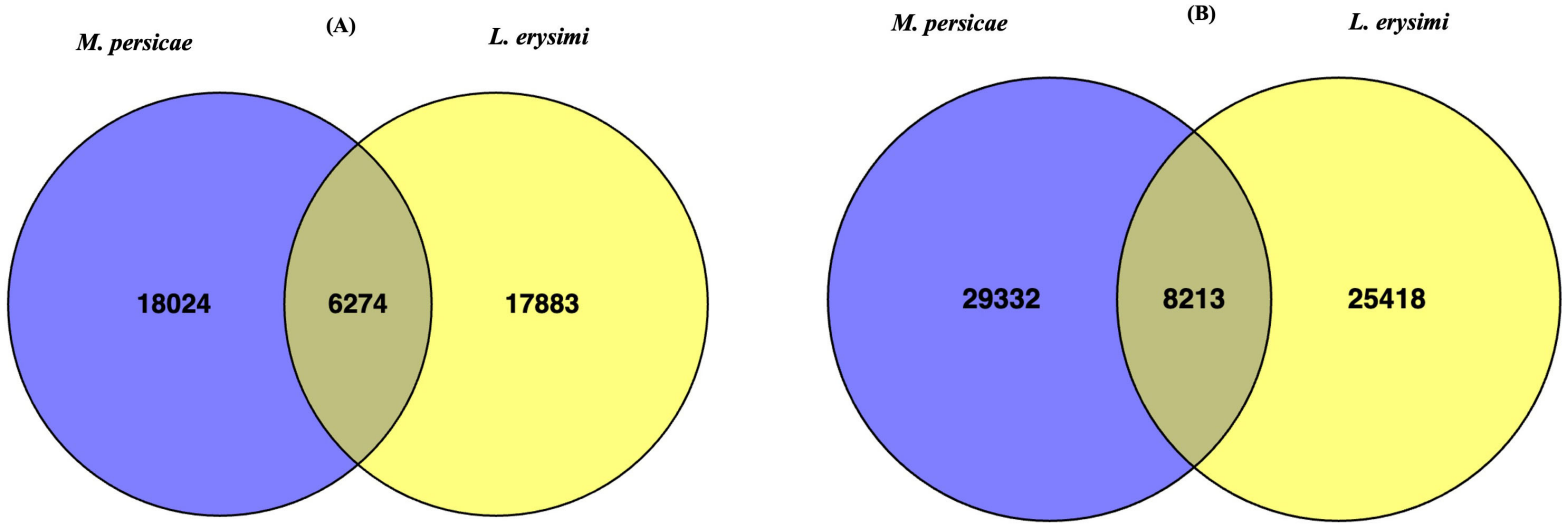
Venn diagram showing unique and commonly expressed unigenes between *M. persicae* and *L. erysimi* at **(A)** adult and **(B)** nymph stages.

Aphid infestation is associated with variations in expression of genes involved with important functions such as host plant recognition, degradation of plant cell wall, detoxification, digestion, and metabolism (Simon et al., 2015; Silva-Sanzana et al., 2020). These categories of genes were identified in both the generalist and specialist aphids in adult as well as nymph stages (**Figure 2**). Earlier studies (Cristofoletti et al., 2003; Pyati et al., 2011; Roy et al., 2016; Hilliou et al., 2021) described limited number of categories such as detoxification enzymes, digestice enzymes, transporters, immunity but did not specifically focus on the variations between a specialist and a generalist. Therefore, our study aims to highlight the key differences among these two categories of aphid species and their infestation and feeding strategies. Hence, we classified all the unigenes obtained in the current into nine categories *viz*., chemosensation-related, plant cell-wall degrading enzymes, detoxification-related genes, digestive enzymes, peptidases, reproduction-related, carbohydrate metabolism, lipid metabolism and amino acid metabolism, all of which play an important role during aphid infestation on host plants. For most functional categories, the number of unigenes was higher in *L. erysimi* as compared to *M. persicae*. However, a significantly higher number of transcripts under the detoxification-related genes category was obtained in adults of *M. persicae* (409) as compared to *L. erysimi* (365) (**Figure 2A**). This category of genes may play an important role in extending the host range of the generalist aphid by allowing neutralisation of a wider repertoire of defence-related secondary metabolites produced by a host plant. Higher number of unigenes were also detected in Plant Cell Wall Degrading Enzymes (PCWDEs) category at nymph stage (**Figure 2B**). Unigenes identified in *L. erysimi* and *M. persicae* at adult and nymph stages were categorised into transcripts that are unique for each aphid and those which are commonly expressed in both species. The generalist, *M. persicae* had greater number of unique transcripts at both developmental stages as compared to the specialist, *L. erysimi* (**Figure 3**). All the predicted unigenes of *L. erysimi* and *M. persicae* were subjected to gene ontology analysis. Maximum number of unigenes were majorly involved in protein binding, metabolic processes and in different cellular entities (**Supplementary Figure 1**).

### Differential expression of genes between generalist and specialist aphids

**Figure 4:**
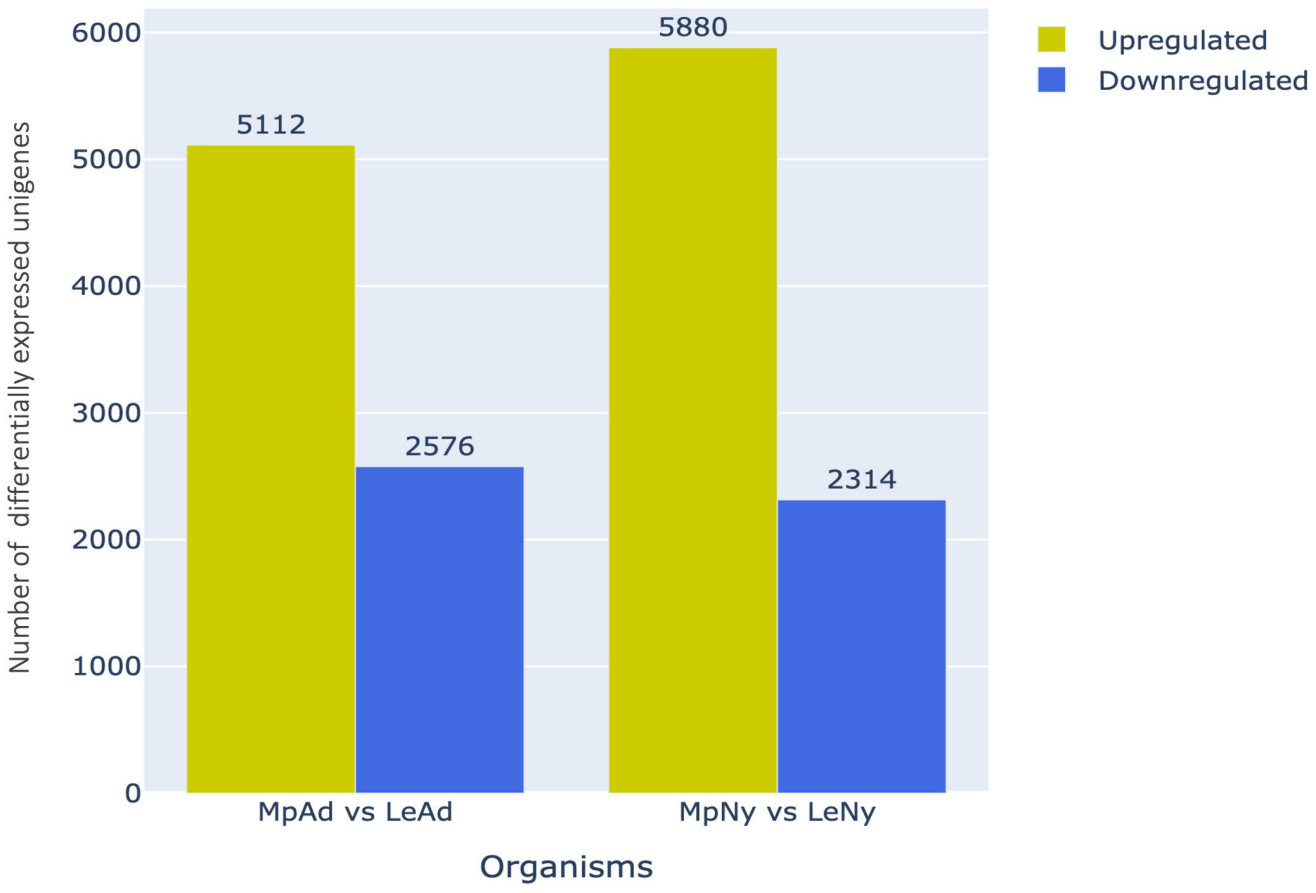
Number of differentially regulated unigenes (upregulated/downregulated) in *M. persicae* relative to *L. erysimi* (Mp vs Le) at adult (Ad) and nymph (Ny) stages.

To identify genes potentially contributing to the generalist or specialist nature of an aphid, we performed differential expression analysis to identify transcripts that showed variations in expression levels between the two aphid species. This analysis resulted in the identification of 15882 DEGs between *M. persicae* and *L. erysimi* of which, 7688 DEGs were identified in the adult stage and 8194 DEGs were identified in nymphs. Of the 7688 differentially regulated adult unigenes, 5112 and 2576 unigenes were upregulated and downregulated respectively in *M. persicae* relative to *L. erysimi* (**Figure 4**). Similarly, in nymphs, 5880 unigenes were upregulated and 2314 unigenes were downregulated in *M. persicae* relative to *L. erysimi* (**Figure 4**). These observations indicate that the generalist aphid has significantly higher levels of expression in a greater number of unigenes relative to the specialist.

Among the differentially expressed transcripts, 92.6% of adult transcripts and 93.9% of nymph transcripts could be annotated using at least one of the databases viz., RefSeq, KEGG pathways, Gene Ontology (GO), Pfam and E.C. number databases (**Supplementary Figure 2**). Among the differentially expressed unigenes, 244 adult and 194 nymph unigenes could be functionally annotated with all the above databases (**Supplementary Figure 2**). Among the DEGs identified between the generalist and specialist adults, 572 unigenes remained un-annotated and 131 unigenes were annotated as ‘uncharacterised proteins’. Among the nymph DEGs, 499 unigenes remained unannotated and 94 DEGs were uncharacterised proteins. The species-based distribution of annotated DEGs against Nr and aphidbase databases are depicted in **Supplementary Figure 3** indicating maximum correspondence to *M. persicae* (35.4% in adults and 40% in nymphs).

### Gene ontology, pFAM and, KEGG pathway annotation of DEGs

According to Gene Ontology classification, 5004 and 5306 differentially expressed unigenes at adult and nymph stages, respectively were categorised into biological process, cellular components, and molecular functions. The distribution of top 15 GO classes indicated that the categories of upregulated and downregulated unigenes were similar in both adults and nymphs (**Supplementary Figure 4**). However, the category of ‘oxidoreductases’, which also includes unigenes involved in detoxification, was unique to the upregulated category of unigenes in *M. persicae* adults and absent in *L. erysimi* adults indicating a more robust response of the generalist aphid in detoxification. Another category of ‘protein synthesis or translation’ in molecular function also included a higher number of up-regulated unigenes at both the stages relative to down-regulated unigenes in *M. persicae* relative to *L. erysimi* (**Supplementary Figure 4**).

To infer differences, if any, in enzymatic functions between the generalist and specialist aphids, E.C. numbers along with the nomenclature of differentially expressed unigenes between *M. persicae* and *L. erysimi* were studied and a total of 1809 and 1948 unigenes were assigned E.C. numbers in adult and nymph stages, respectively (**Supplementary Figure 5**). The top 15 enzyme categories included those that play an important role during aphid feeding and combating host defences. Examples include oxidoreductases, hydrolases (Sadek et al., 2013), peptidases and enzymes involved in oxidation-reduction reactions. Our data indicates a significantly higher number of upregulated unigenes in these categories in the generalist aphid as compared to the specialist. Further, higher expression levels of these unigenes in the generalist aphid indicates a more vigorous system of sequestering secondary metabolites produced by host plants against insect herbivory at both developmental stages when compared to the specialist aphid.

Differentially expressed unigenes between the two aphid species across different biochemical pathways assigned using the KEGG database were also studied. In total, 1473 differentially expressed unigenes were assigned 33 KEGG pathways at the adult stage and 31 pathways were assigned to 957 differentially expressed unigenes at the nymph stage of both the species. The top 15 differentially expressed pathways between the generalist and the specialist aphid at both the stages (**Supplementary Figure 6**) included metabolic pathways, carbohydrate metabolism, signal transduction, xenobiotics biodegradation and immune system.

### Classification of DEGs into categories of interest

**Figure 5:**
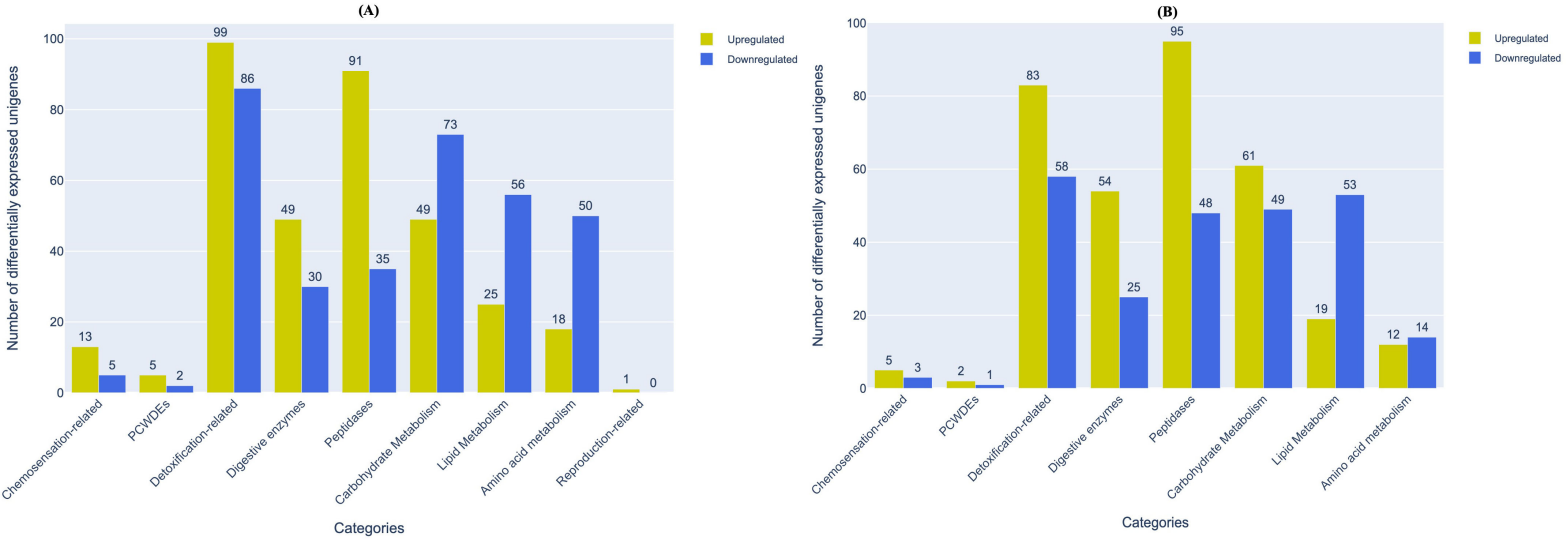
Distribution of differentially expressed unigenes between *M. persicae* and *L. erysimi* in important functional categories of interest in **(A)** adults and **(B)** nymphs. Numerals above the bars indicate the number of genes in that category in each aphid species.

From host recognition to initiation of the feeding process, aphids undergo various physiological changes related to multiple processes *viz*., olfactory signal transduction, sap-sucking, detoxification, digestion, combating host defences and procreation (Powell et al., 2006; Simon et al., 2015; Wang et al., 2020b). Concurrently, they also alter the host system by eliciting and/or curbing defence responses (Giordanengo et al., 2010; Elzinga and Jander, 2013; Mondal, 2017). We had earlier categorised the unigenes of *M. persicae* and *L. erysimi* into nine functionally important categories associated with the above processes and reported variations in the number of unigenes in each category between the two aphid species (**Figure 2**). Based on differential expression data from our transcriptomes, we analysed the number and distribution of differentially expressed genes in each of these nine categories (**Supplementary File 1**). Our data indicates that in almost all categories of interest except carbohydrate metabolism, lipid metabolism and amino acid metabolism, the generalist aphid, *M. persicae* had greater number of unigenes that showed significantly higher expression levels compared to the specialist, *L. erysimi* (**Figure 5**). These unigenes might be important candidate genes associated with the generalist nature of *M. persicae*.

**Figure 6:**
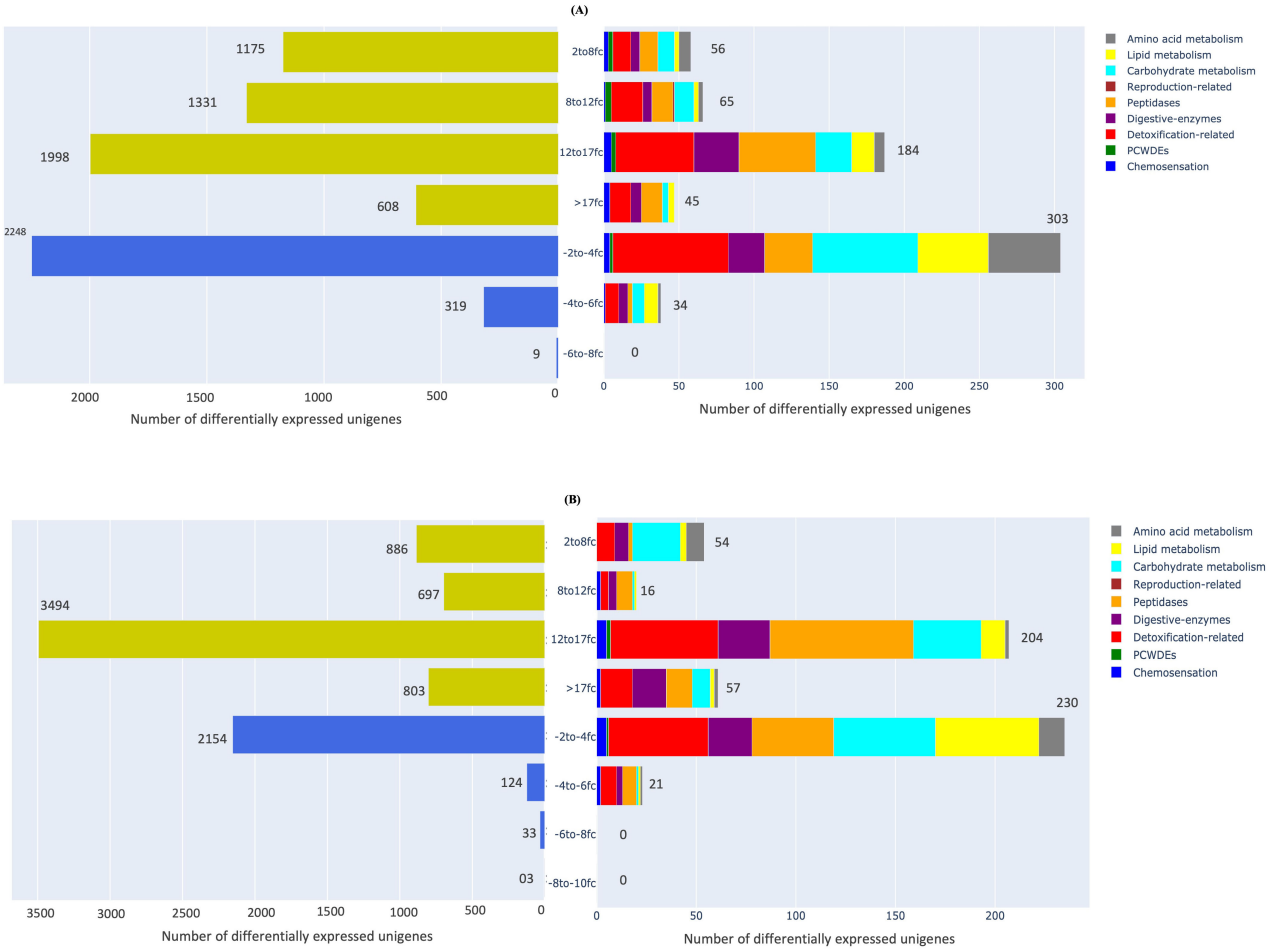
Distribution of differentially expressed unigenes between *M. persicae* and *L. erysimi* adults and nymphs in different ranges of log2-fold changes (fc). Yellow-coloured bars of the graph on the left-hand side represent upregulated range in *M. persicae* relative to *L. erysimi* whereas blue-coloured bars represent downregulated range in **(A)** adults and **(B)** nymphs. Bar graph on the right-hand side of the figure represents different ranges of fold change among genes specific to the nine functionally important categories in **(A)** adults **(B)** nymphs.

We also analysed the distribution of genes among the nine important functional categories of interest vis-à-vis their fold-change in expression in both adults and nymphs (**Figure 6**). Those unigenes which showed differential expression in *M. persicae* at both developmental stages ranged from 2- to >17 log2-fold changes while the corresponding range for downregulation was -2 to -8 log2-fold changes relative to *L. erysimi*. This indicated that the range of upregulation in the generalist, *M. persicae* was significantly higher than that observed in the specialist, *L. erysimi*. In *M. persicae* adults, maximum number of unigenes (1998) showed an upregulation in the 12- to 17 log2-fold range. We observed a -2- to -4 log2-fold range reduction in expression levels for 2248 genes in *M. persicae* (**Figure 6**). Similar trends were observed in nymphs for both species with 3494 genes of *M. persicae* being upregulated in the 12- to 17 log2-fold range and 2154 genes were downregulated in the 2- to 4- log2-fold range when compared to *L. erysimi*. A significantly high number of genes (608 in adults and 803 in nymphs) were in the maximum range of upregulation (>17 log2-folds) in *M. persicae*. Many differentially regulated unigenes from both adults and nymphs could not be annotated (**Supplementary Table 2**). All the un-annotated unigenes which are differentially expressed at higher fold-changes could be novel candidate genes which might be functionally associated with the generalist nature of *M. persicae* and/or specialist behaviour of *L. erysimi*. These candidate genes would need to be studied further to understand their functional role(s), if any, in the biology of both species.

### Variations in host recognition and selection genes

**Figure 7:**
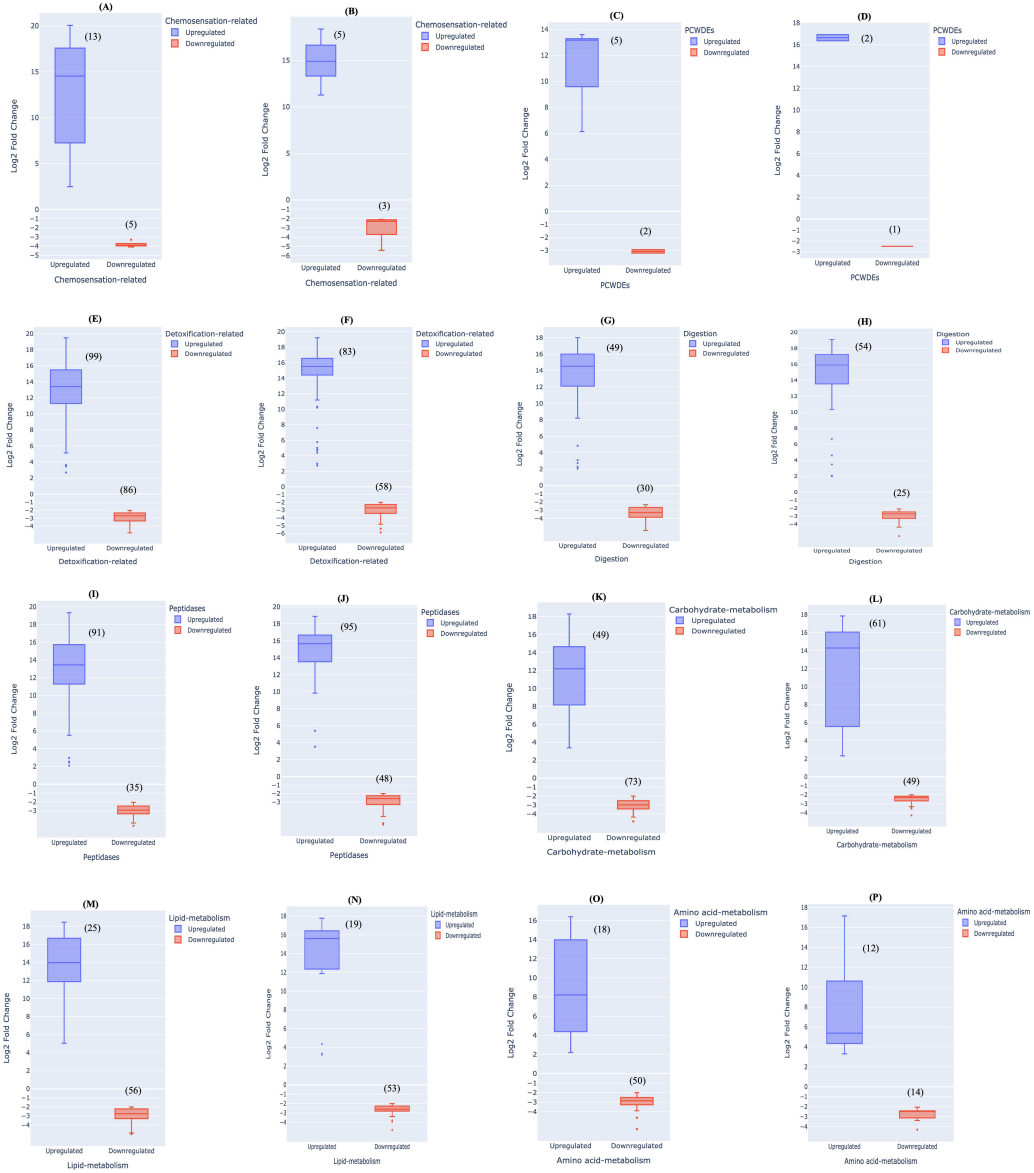
Range of upregulation and downregulation of differentially expressed unigenes between *M. persicae* and *L. erysimi* at adult stage in different categories- Chemosensation-related **(A)** adults **(B)** nymphs; plant cell wall modifying enzymes (PCWMEs) **(C)** adults **(D)** nymphs; detoxification-related **(E)** adults **(F)** nymphs; digestive-enzymes **(G)** adults **(H)** nymphs; peptidases **(I)** adults **(J)** nymphs; carbohydrate-metabolism **(K)** adults **(L)** nymphs; lipid-metabolism **(M)** adults **(N)** nymphs; amino-acid metabolism **(O)** adults **(P)** nymphs.

To settle on a host plant, it is important for herbivorous insects to perceive cues related to a suitable host. This is achieved by activating a complex chemosensory pathway of host recognition that includes various kinds of proteins like odorant binding receptor/protein, pheromone-binding protein, olfactory receptors, gustatory receptors and chemosensory receptors (Zhang et al., 2020; Wang et al., 2020b; Huang et al., 2023; Powell et al., 2006). An earlier study by He et al. (2023) revealed the presence of fewer chemosensory genes, odorant binding proteins and gustatory receptors in *Schlechtendalia chinensis*, a specialist aphid in contrast to *Myzus persicae*. A lower number of chemosensory genes in a specialist aphid may be the cause of its narrow host range requiring fewer host recognition genes as compared to the generalist aphid. Likewise in our study, among the differentially expressed unigenes between *M. persicae* and *L. erysimi*, ‘chemosensation-related’ properties were present in 18 and 8 unigenes at adult and nymph stages, respectively. Among these, 13 unigenes showed higher expression in the range of 2- to 20- log2-fold changes relative to *L. erysimi* and 5 genes were downregulated in adults of *M. persicae* (**Figure 7A**). Similarly, 5 unigenes showed higher expression in *M. persicae* nymphs as compared to *L. erysimi* ranging from 11- to 18- log2-fold changes while 3 unigenes were downregulated in range of -2 to -5 log2-fold changes (**Figure 7B**). Generalist aphids are considered to be more flexible in their host preferences and these differences with the specialist could influence their search for appropriate hosts (Tapia et al., 2015). Therefore, upregulation of a larger number of unigenes involved in ‘chemosensation’ in *M. persicae* might indicate a more robust, active and diversified host-recognition mechanisms in the generalist aphid as compared to the specialist.

### Variations in plant cell wall degrading enzyme genes

Aphid saliva contains PCWDEs which facilitate stylet penetration into host tissues. These enzymes are important for the infestation process and also play a role in eliciting plant defence responses (Silva-Sanzana et al., 2020). Studies conducted on aphids and related hemipterans have identified cell-wall degenerating enzymatic activities in their saliva. Pectinase and cellulase activity have been observed in the saliva of *S. avenae* (Guangxi et al., 2006) while a transcriptome study on *M. persicae* reported the presence of cellulase transcripts (Thorpe et al., 2016). In our study, we identified differential expressed unigenes for PCWDEs including pectinacetylesterases, glucanases, glucosidases and beta-mannosidase, all of which are responsible for cell wall degradation and aid in stylet penetration (Zhang et al., 2023). In the generalist aphid, 5 and 2 unigenes were upregulated with 10- to 13- log2 fold-changes and 13 log2 fold changes at adult and nymph stages, respectively (**Figures 7C, D**). Downregulation in expression levels was observed in two unigenes with -2 to -3 log2 fold-changes in *M. persicae* adults and at nymph stage, one unigene was downregulated as compared to *L. erysimi* (**Figures 7C, D**). In both developmental stages of *M. persicae*, we observed a greater number of upregulated unigenes with higher fold changes as compared to *L. erysimi* that may provide a greater ability to the generalist aphid for altering the cell wall of host plant as compared to the specialist. Furthermore, since a generalist aphid has a broader host range, involvement of more number of cell wall-altering genes may further assist the generalist aphid to commence the penetration and sap-testing process more rapidly as compared to the specialist.

### Variations in detoxification-related genes

Selection of host plants by aphids is influenced by the diversity of plant metabolites. While a specialist aphid chooses the host based on few metabolites (mainly secondary metabolites), a generalist aphid screens a much larger scale of both primary and secondary metabolites (Kennedy and Booth, 1951; Bernays, 2001; Powell et al., 2006). Secondary metabolites released by host plants are an important component of plant defence against insect herbivory. Moreover, these metabolites also have antixenotic properties that either repel aphids or attract their natural enemies (Wagner et al., 2004; Walling, 2008; Sadek et al., 2013). Insects respond to such metabolites by releasing detoxifying enzymes and oxidoreductases (Cai et al., 2009). In a study by Ramsey et al. (2010), comparison of transcriptome data generated for a generalist, *M. persicae* (green peach aphid) with data available for a specialist, *A. pisum* (pea aphid) indicated 40% higher number of cytochrome P450 detoxifying enzyme genes in the generalist compared to the specialist. No significant differences were detected in other enzymes between the two species. In our study, we identified 185 and 141 detoxification-related, differentially expressed unigenes in adult and nymph stages, respectively between *M. persicae* and *L. erysimi*. Examples of such genes include catalase, superoxide dismutase, cytochrome P450, glutathione S- transferase, peroxidase, UDP- glucuronosyltransferase, genes involved in cellular oxidant detoxification, oxidoreductase activity and monooxygenase activity (Sadek et al., 2013; Wang et al., 2020a; Durak et al., 2021; Hilliou et al., 2021). Of all the differentially regulated unigenes between *L. erysimi* and *M. persicae* that might be involved in detoxification process, 99 and 83 unigenes showed significantly higher expression levels in adults and nymphs, respectively of *M. persicae*. Further, these genes had substantial log2-fold changes of 2- to 19- log2 fold changes in adults and 4- to 18- log2 fold changes in nymphs, respectively. On the other hand, 86 and 58 unigenes showed downregulation of -2 to -4 and -2 to -5 log2 fold changes in adults and nymphs, respectively (**Figures 7E, F**). These results indicate that the extent of upregulation of detoxification genes in *M. persicae* was significantly higher than the downregulation levels of detoxification genes in *M. persicae*. A greater number of unigenes involved in detoxification and more importantly, a much higher extent of up-regulation in *M. persicae* might be attributed to the fact that *M. persicae* being a generalist, is exposed to varied types of plant defensive metabolites. To counter a larger repertoire of plant metabolites and overcome challenges like plant toxins and digestive inhibitors, generalists may have to adopt a wider and a more robust system for detoxification (Bansal and Michel, 2018). On the other hand, specialists are well-adapted to their host plants and therefore might have a limited but streamlined detoxification system suitable for neutralising specific metabolites of their host plants. This was demonstrated in a study by Govind et al. (2010) on a wild type and a defence-less transgenic plant of *Nicotiana attenuata*, wherein the specialist herbivore *(Manduca sexta)*, being adapted to its host metabolite, nicotine, regulated only specific detoxifying genes according to the defence response of the host plant. However, the generalist herbivore *(Heliothis virescens)* continued to generate a robust response even against the defence-less transgenic plant i.e., irrespective of the status of its host plant.

### Variations in digestion-related genes

Differentially expressed unigenes involved as digestive enzymes were included in this category. This category comprises cathepsin, maltase, aminopeptidase, trehalase, alpha-glucosidase and trypsin (**Figures 7G, H**). In this category too, *M. persicae* had a larger number of significantly upregulated unigenes with 49 and 54 unigenes (including variants of the above enzymes) being upregulated in adults and nymphs, respectively. The upregulation varied from 2- to 18- fold2 change and 2- to 19- log2 fold change in adults and nymphs, respectively (**Figures 7G, H**).The downregulated unigenes included 30 and 25 unigenes with a range of -2 to -5 log2 fold change and -2 to -4 log2 fold change in adults and nymphs, respectively (**Figures 7G, H**). In our study more number of upregulated genes were observed in all categories of digestive enzymes (except Maltase) in both adults and nymphs of *M. persicae* as compared to *L. erysimi* (**Supplementary Figure 7**). Due to narrower host range, a specialist aphid might require fewer specialised enzymes to process chemical compounds present in their diet. On the other hand, our results also indicate that polyphagy shown by a generalist aphid requires a broader range of adaptations to digest varied components of diet from multiple hosts and correlates with the study of Roy et al. (2016).

### Variations in proteolysis-related genes

In addition to high sugar content, phloem sap also contains proteins that can vary between 0.3 to 60mg/ml depending upon the host species (Pyati et al., 2011). It was earlier believed that ingested plant-sap does not undergo proteolysis in hemipterans. However, recent studies have shown that proteolysis in sap-feeding insects is essential for their proper nutrition (Salvucci et al., 1998; Foissac et al., 2002; Cristofoletti et al., 2003). The category of ‘Peptidases’ comprised of all unigenes that have enzymatic action on peptide bonds and included a total of 126 and 143 differentially expressed unigenes between *L. erysimi* and *M. persicae* at adult and nymph stage. Of these, 91 and 95 unigenes were upregulated in *M. persicae* at adult and nymph stages, respectively while 35 and 48 unigenes showed lower levels of expression in adults and nymph stages, respectively (**Figures 7I, J**). Upregulation of these differentially expressed unigenes ranged from 2- to 19- log2 fold change and 3- to 19- log2 fold changes in adults and nymphs, respectively (**Figures 7I, J**). On the other hand, downregulation ranged from -2 to -4 log2 fold changes and -2 to -6 log2 fold changes in adults and nymphs, respectively (**Figures 7I, J**). Phloem sap ingested by aphids also contains many proteins with anti-insect properties as a component of plant defence (Kehr, 2006). Consequently, peptidases allow aphids to neutralise the effects of these defence proteins to facilitate infestation of the host plant. The presence of a larger number and an increased level of upregulation of peptidases in *M. persicae* may be advantageous in broadening its host range and allowing rapid infestation.

### Variations in reproduction-related genes

As soon as favourable conditions are available during the growing season of *Brassica juncea*, both *M. persicae* and *L. erysimi* become prevalent. In order to establish its population on the host plant, the aphids need to expedite the process of feeding and reproduction. In case of a generalist aphid, higher number/expression of reproduction-related genes would allow rapid establishment of these aphids on a broad range of host plants. Interestingly, our study identified more number of reproduction-related genes in *L. erysimi* as compared to *M. persicae* (**Figure 2**). However, in consonance with the above hypothesis, one unigene related to ‘reproduction-process’ had significantly higher expression levels in *M. persicae* adults (more than 11 log2 fold change) as compared to adults of *L. erysimi*. None of the other reproduction-related genes showed differential expression between the two aphid species. It would be interesting to study the functional role of this gene in aphid biology and ascertain its contribution, if any, to the increased occurrence, fecundity, persistence and distribution of the generalist aphid on host plants, including *B. juncea*.

### Variations in metabolism-related genes

Categories such as metabolism of carbohydrates, lipids and amino acids included a total of 122, 81 and 68 differentially regulated unigenes, respectively between *M. persicae* and *L. erysimi* adults (**Figures 7K, M, O**). The corresponding numbers in nymphs were 110, 72, 26 differentially regulated unigenes (**Figures 7L, N, P**). In these categories, most differentially expressed unigenes showed significantly lower expression levels in *M. persicae* at both the developmental stages in the range of -2 to -6 log2 fold changes in adults and nymphs. Though the number of upregulated unigenes in *M. persicae* was lower than that of *L. erysimi* in all categories except carbohydrate metabolism in nymphs (**Figure 5**), the extent of upregulation was much higher (from 2- to >17 log2 fold change) in the generalist aphid indicating an increased ability to digest these categories of molecules from phloem sap. However, greater number of upregulated unigenes in *L. erysimi* might be an indication that specialists are capable of processing their diet from specific host plant more efficiently and completely than generalists. Since generalists have the option to feed upon multiple hosts, they might have a more generalised mechanism of metabolism for all kinds of host plants which may further lead to incomplete digestion of metabolites (Olazcuaga et al., 2023).

Our study was targeted towards studying variations in the molecular responses displayed by a generalist aphid and a specialist aphid growing under the same conditions and feeding on the same host plant i.e., *Brassica juncea* var. Varuna. It allowed analysis of the similarities and differences in expression of genes involved in aphid-plant interactions and more importantly, it facilitated identification of differences in the responses of the generalist and the specialist aphid against defence mechanisms of the host. It generated an exhaustive repertoire of genes in the generalist and specialist aphids and allowed their comparison between the corresponding adult and nymph developmental stages. To further validate and substantiate the identified trends in our study between generalist and specialist aphids, further studies can include a larger number of generalist aphids with a broader host range and corresponding specialists, which would provide more comprehensive information about the molecular signatures that confer advantage to generalist aphids and the evolutionary mechanisms.

### Identification of effectors and their variations between generalist and specialist aphids

**Figure 8:**
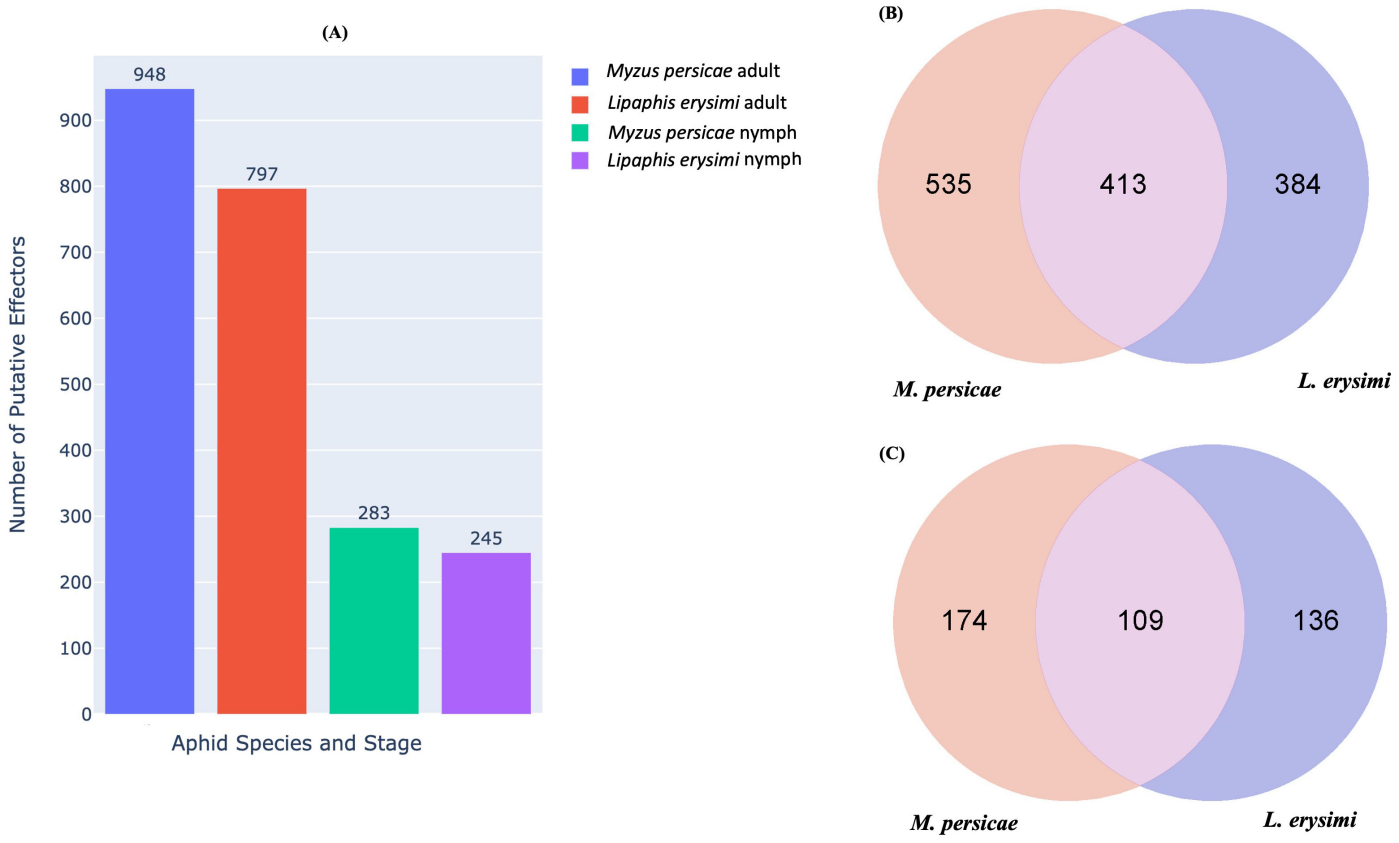
**(A)** Number of putative effectors predicted in *M. persicae* and *L. erysimi* at adult and nymph stages. Venn diagram representing the number of common and unique putative effectors between *M. persicae* and *L. erysimi* in **(B)** adults and **(C)** nymphs.

Aphids, while probing and feeding on host plants, release effector molecules many of which, are species-specific (Elzinga and Jander, 2013). Effectors play a major role in aphid-plant interactions including host recognition (Mondal, 2017, 2020). Many effectors are recognised by host plants which then initiate defence responses against the insect and this effectors-based interaction determines the consequences of aphid and host plant interactions (Bos et al., 2010; Elzinga and Jander, 2013; Jaouannet et al., 2014; Mondal, 2017). *In silico* identification of putative effectors identified 948 effectors in adults and 283 effectors in nymphs of *M. persicae* while in *L. erysimi*, 797 and 245 putative effectors showed expression at adult and nymph stages, respectively (**Figure 8A**). Among the identified effectors, 535 and 174 effectors were unique to *M. persicae* adults and nymphs, respectively while the corresponding figures for *L. erysimi* adults and nymphs were 384 and 137, respectively (**Figures 8B, C**). The larger repertoire of effectors, including a higher number of unique effectors in the generalist aphid compared to the specialist might facilitate feeding, modulate hosts’ defence responses more effectively and more importantly, confer on the generalist, an ability to infest multiple hosts.

Many putative effectors identified in the current study showed homology with effectors of other aphid species available in public domain. Protein sequences of 1094 published effectors of *A. pisum*, *M. persicae* and *D. noxia* (Carolan et al., 2011; Bos et al., 2010; Nicolis et al., 2022) were obtained using transdecoder. Following manual curation, a total of 1044 protein sequences were retrieved and used to perform BlastP analysis with identified putative effectors of adults and nymphs of both species. Among adults, out of 1403 identified putative effectors, 854 (60.86%) showed homology with the published effectors. Of the 420 effectors detected in nymphs, 281 (66.9%) were found to be homologous to effectors of other aphid species. Most effectors identified in our study [652 (46.47%) in adults and 220 (52.38%) in nymphs] were homologous to *D. noxia*. The remaining effectors showed homology with *A. pisum* and *M. persicae*. (**Supplementary File 2**).

Our study also identified several effectors from both species which did not show any homology with above-mentioned published data of effectors. In *M. persicae*, out of 535 and 174 uniquely expressed putative effectors in adults and nymphs, 190 and 62 putative effectors, respectively did not show any homology with effectors of other three species. Similarly, in *L. erysimi*, out of 384 and 137 uniquely expressed putative effectors at adult and nymph stages, 176 and 54 effectors showed no homology with other effectors. These effectors can be studied further for their functional roles which might allow identification of molecular signatures that govern variations between the generalist and specialist aphids.

Annotation of putative effectors identified in our study against multiple databases resulted in annotation of 95.2% and 100% of adult and nymph effectors, respectively. Significant progress has been achieved towards understanding the potential functions of many effectors. Some of them have been functionally characterised and have been used as candidates for introducing aphid resistance in agricultural crops. For example, *c002*, an effector protein that was first described in *A. pisum* (Mutti et al., 2006, 2008) was shown to be essential for successful and prolonged feeding on the host plant. Overexpression of *M. persicae c002* was found to promote aphid fecundity and proliferation on the host plant (Bos et al., 2010; Pitino et al., 2011). Putative effectors identified from our transcriptome data include antioxidant and detoxifying enzymes such as superoxide dismutase, peroxidase, glucose dehydrogenase and some effectors aiding the feeding process and manipulating host defence and immunity viz., odorant-binding proteins, calcium-ion binding, insect pheromone-binding and chemosensory protein. The identified effectors also included digestive enzymes such as maltase, cathepsin-B and L, aminopeptidase, trypsin, serine protease and a few plant cell wall modulating enzymes like beta-mannosidase and glucosidase II beta subunit. Sheath proteins such as mucin-2 and mucin-5AC were also identified in adults and nymphs, respectively. We also detected ARMET, a Ca^2+^-binding salivary protein which is known to interfere in Ca^2+^trafficking from ER- membrane to the sieve element. Suppression of ARMET had a negative impact on the feeding and life span of aphids (Wang et al., 2015; Hafke et al., 2009).

To analyse variations, if any, in the functional repertoire of effectors between generalist and specialist aphids, we studied their annotations from both aphids at adult and nymph stages. Adults of *M. persicae* showed a significantly greater number of effectors under the categories of ‘detoxification-related’ and ‘digestive enzymes’ (**Table 2**). The generalist aphid thus appears to demonstrate a stronger response in neutralising host defence responses and digesting components acquired during feeding through its effectors, both of which could play an important role in widening its host range. Adults of *L. erysimi* showed more number of putative effectors related to host recognition (chemosensation-related) and onset of feeding process (PCWDEs) (**Table 2**). In case of nymphs, higher number of ‘detoxification-related’ effectors were observed in *L. erysimi* while nymphs of *M. persicae* had more number of effectors in ‘PCWDEs’ and ‘digestive enzymes’ categories (**Table 2**). Thus, we may conclude that effectors in the generalist adults are more active in detoxification and digestion while *L. erysimi*, being a specialist, has more effectors that aid in host localisation and also in interrupting the first line of host defence i.e. plant cell wall.

**Table 2:** Number of total and unique effectors in *M. persicae* and *L. erysimi* under different categories of interest at adult and nymph stage.

Categories of interest	Total number of putative effectors in *M. persicae*	Total number of putative effectors in *L. erysimi*	Number of unique effectors in *M. persicae*	Number of unique effectors in *L. erysimi*
Adults				
Chemosensation-related	9	13	1	5
PCWDEs*	3	5	1	3
Detoxification-related	21	11	18	8
Digestive enzymes	64	37	45	18
Nymphs				
Chemosensation-related	6	6	5	5
PCWDEs*	1	0	1	0
Detoxification-related	2	4	2	4
Digestive enzymes	13	8	6	1

*PCDWEs, Plant Cell Wall Degrading Enzymes.

**Figure 9:**
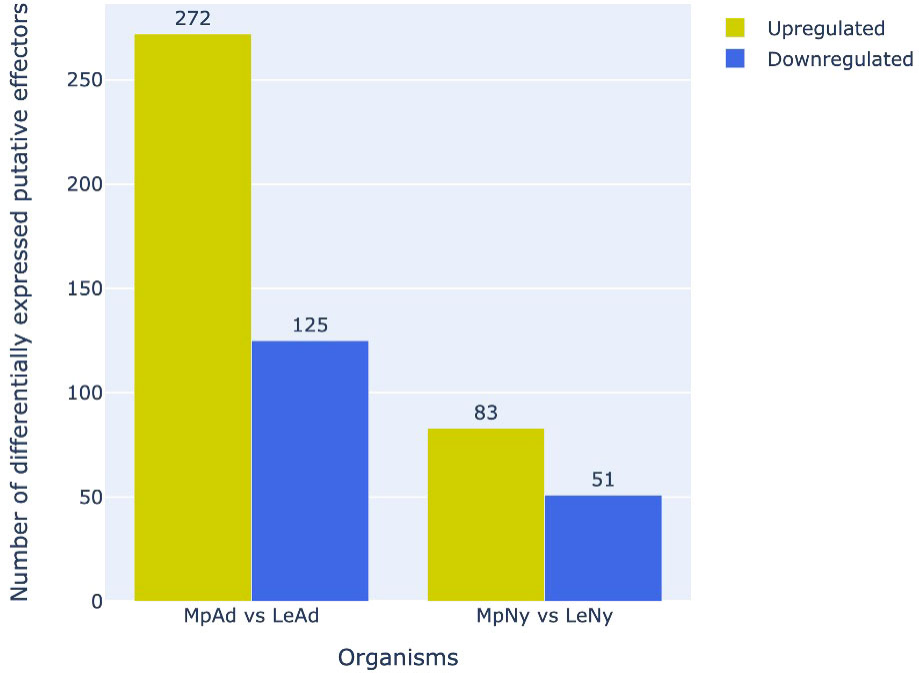
Number of differentially regulated putative effectors (upregulated/downregulated) in *M. persicae* relative to *L. erysimi* (Mp vs Le) at adult (Ad) and nymph (Ny) stages. Numerals above the bars indicate the number of genes in that category in each aphid species.

Analysis of differential expression profiles of putative effectors between *L. erysimi* and *M. persicae* indicated that a significantly higher number of effectors were upregulated in *M. persicae* in both adult and nymph stages as compared to *L. erysimi* (**Figure 9**). Interestingly, as many as 10 detoxification-related genes that were common to both the aphids were found to be significantly upregulated in the generalist aphid. A list of all the differentially expressed effectors along with their functional annotation is provided in **Supplementary File 2**. These results indicate that, in addition to the higher number of effectors, generalist aphids also have significant up-regulation of such genes (including those in important functional categories) as compared to the specialist. These molecular signatures could play an important role in determining increased efficacy of the generalist aphid in infesting a large number and wider range of host plants.

### Validation of differentially expressed unigenes by qRT-PCR

**Figure 10:**
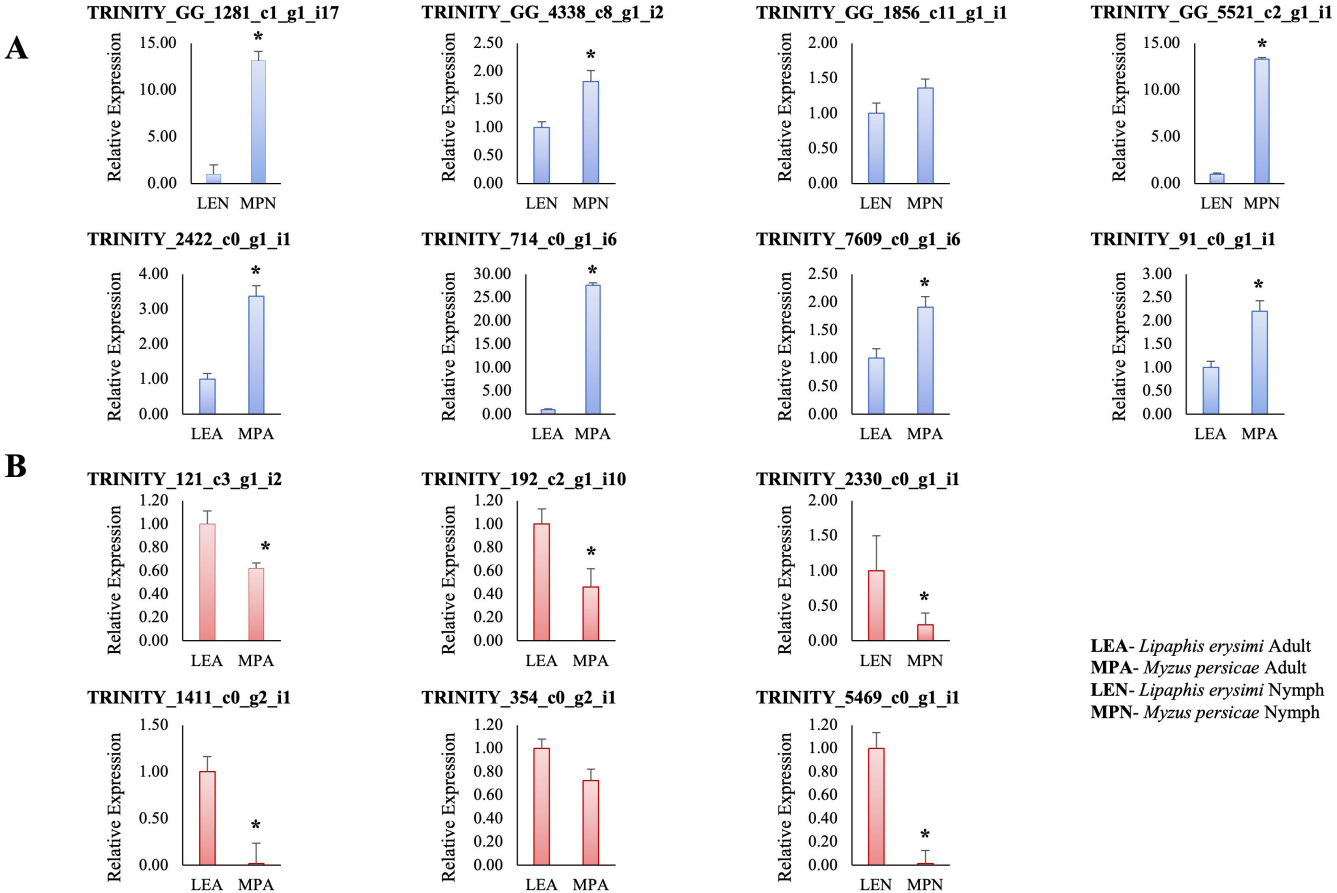
Graphs representing the relative expression of 14 differentially expressed unigenes between *Myzus persicae* adult (MPA) and *Lipaphis erysimi* adult (LEA); *Myzus persicae* nymph (MPN) and *Lipaphis erysimi* nymph (LEN). **(A)** represents the transcripts which are upregulated in *Myzus persicae* (downregulated in *Lipaphis erysimi*) with respect to *Lipaphis erysimi* at both adult and nymph stages in transcriptome data. Among the upregulated transcripts are TRINITY_2422_c0_g1_i1- involved in Reproduction process, TRINITY_GG_1281_c1_g1_i17- plasma membrane calcium-transporting ATPase 2 isoform X1, TRINITY_GG_1856_c11_g1_i1-dihydrolipoyl dehydrogenase, TRINITY_GG_4338_c8_g1_i2- capon-like protein isoform X2, TRINITY_DN714_c0_g1_i6- uncharacterised protein. Transcripts-TRINITY_GG_5521_c2_g1_i1- protein disulfide-isomerase A3 and TRINITY_7609_c1_g1_i1-unannotated, are upregulated putative effectors which do not show any homology with other species’ effector sequence. **(B)** represents transcripts which are downregulated in *Myzus persicae* (upregulated in *Lipaphis erysimi*) with respect to *Lipaphis erysimi* at both adult and nymph stages in transcriptome data. Among the downregulated transcripts are, TRINITY_DN354_c0_g2_i1- repetitive proline-rich cell wall protein 2-like, TRINITY_DN1411_c0_g2_i1- cuticular protein 41 precursor, TRINITY_DN5469_c0_g1_i1- cathepsin B-like cysteine proteinase 4 isoform X2, TRINITY_DN192_c2_g1_i10- DNA-directed RNA polymerase II subunit, TRINITY_DN2330_c0_g1_i1- protein yellow. TRINITY_DN121_c3_g1_i2- unannotated, is a downregulated putative effector which does not show any homology with other species’ effector sequence. Asterisks present above the bars in graphs show statistically significant differences between the two samples (p_value_ <0.05).

For validation of expression levels of transcripts by qRT-PCR, 14 differentially expressed transcripts with fold changes of >2 to <2 at p_adjusted_ value <0.05 were selected which also included differentially regulated putative effectors (which did not show homology with other organisms). Of these 14 transcripts, 12 transcripts showed statistically significant results at p_value_<0.05 and the remaining two transcripts (TRINITY_DN354_c0_g2_i1, TRINITY_GG_4338_c8_g1_i2) showed non-significant results (**Figure 10**). Significantly upregulated transcripts included one transcript (TRINITY_2422_c0_g1_i1) involved in reproductive process and showed high levels of expression in *M. persicae* adults as compared to adults of *L. erysimi*. This gene could be an interesting candidate for further studies on its potential contribution to higher reproductive rates of generalist aphids and could also be a potential target for aphid resistance in crop improvement strategies. Among other upregulated transcripts were two putative effector transcripts (TRINITY_GG_5521_c2_g1_i1, TRINITY_7609_c1_g1_i1). Details of remaining transcripts are provided in **Supplementary File 3**. On the other hand, downregulated transcripts (in *M. persicae* adults and nymphs) included a putative effector transcript (TRINITY_DN121_c3_g1_i2), cuticular protein 14 precursor (TRINITY_DN1411_c0_g2_i1), cathepsin-B (TRINITY_DN5469_c0_g1_i1), protein yellow (TRINITY_DN2330_c0_g1_i1) transcripts and others, details of which are provided in **Supplementary File 3**. qRT-PCR results for all the selected differentially expressed transcripts between *M. persicae* and *L. erysimi* at adult and nymph stages were in consonance with the expression patterns concluded from transcriptome data.

### Ka/Ks analysis

**Figure 11:**
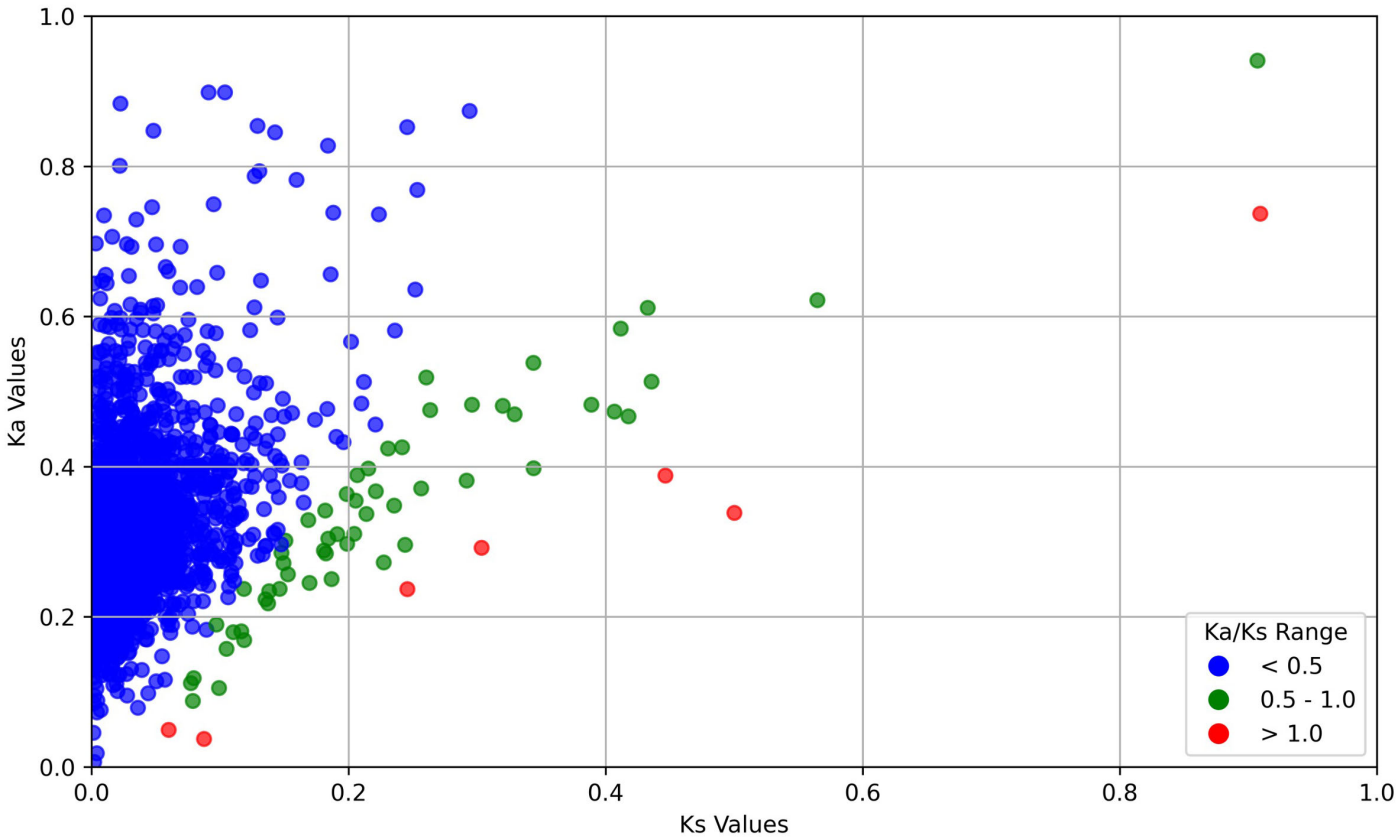
Distribution of Ka and Ks between *M. persicae* and *L. erysimi*. Sequences with Ka/Ks >1 i.e. positive selection are displayed in red colour; sequences with Ka/Ks between 0.5-1.0 are displayed in green and sequences with Ka/Ks less than 0.5 are displayed in blue colour.

In order to study the genes that have contributed significantly towards the evolutionary divergence between the two species, we performed Ka/Ks analysis between orthologous groups of the two species. Ka/Ks ratio is broadly used to provide information about the intensity of evolutionary force and its mode of selection, acting on a coding gene between organisms. Ka/Ks > 1 represents a positive selection, Ka/Ks =1 indicates neutral selection and Ka/Ks < 1 reflects negative selection. To understand whether a gene is undergoing purifying or diversifying selection, rate of substitutions is measured within orthologous pairs of two species. We calculated Ka/Ks ratio between *L. erysimi* and *M. persicae*. From the transcriptome data of *L. erysimi* and *M. persicae*, 2904 one-to-one orthologs were detected of which, 7 pairs of groups had Ka/Ks > 1 indicating that these genes of both aphid species were under positive selection i.e., they are undergoing diversification (**Figure 11**). These 7 pairs of include unigenes involved in lipid metabolism, transcription, protein sumoylation which further can studied to understand their functional relevance in evolutionary divergence. Out of these 7 pairs of genes, one unannotated unigene which was identified as a putative effector showed expression only in *L. erysimi* adults. Three unigenes were differentially expressed between the two aphid species. In the generalist aphid, *M. persicae*, two of these unigenes were upregulated with 14- and 16- log2 fold changes at nymphal stage whereas one unigene was downregulated with -2 log2 fold changes. Swanson et al. (2004), in their study re-adjusted the cut-off value of 1 to 0.5 by showing evidence for evolution of genes with 1> Ka/Ks >0.5. Additionally, many other studies used this new range for signifying positive selection. In our data, we found 67 pairs of one-to-one orthologs of *M. persicae* and *L. erysimi* having 1>Ka/Ks>0.5 and thus, these pairs of genes can also be considered as candidates undergoing positive selection. Among these 67 pairs of unigenes, we found 8 unigenes identified as putative effectors of *L. erysimi* and *M. persicae* which further might be responsible for this diversified feeding behaviour of a specialist aphid versus a generalist aphid.

## Concluding remarks

Variations in expression patterns of genes between the generalist and specialist aphids at two developmental stages revealed significant differences in genes that are involved in various stages of the infestation process. Although the specialist, *L. erysimi* had a greater number of unigenes than the generalist, differential expression studies confirmed significantly higher numbers and levels of upregulation of unigenes in various functionally important categories in the generalist aphid. Similarly, putative effectors that were identified in our study were expressed in greater numbers in the generalist than the specialist. We identified significant variations in genes involved in various stages of the infestation process *viz*., host recognition and selection, plant cell wall degradation, detoxification of plant defence responses, digestion and metabolism between the two aphid species. Our data identifies important candidate genes that govern the robust and polyphagous nature of the generalist aphid. To the best of our knowledge, such a study on analysing variations in gene expression patterns between a generalist and specialist aphid has not been conducted till date. It would serve as an important resource for aphid biologists and facilitate further studies on aphid biology and evolution. Crucifers have a significant economic importance worldwide as a source of vegetables or oilseeds and are severely affected by infestation of both aphid species. Our data builds on the current understanding of aphid biology of these two species and also provides important candidate genes that could be used in crop protection strategies.

## Data Availability

The datasets presented in this study can be found in online repositories. The names of the repository/repositories and accession number(s) can be found below: https://www.ncbi.nlm.nih.gov/, SRR28520211; https://www.ncbi.nlm.nih.gov/, SRR28520210; https://www.ncbi.nlm.nih.gov/, SRR28520207; https://www.ncbi.nlm.nih.gov/, SRR28520206; https://www.ncbi.nlm.nih.gov/, SRR28520205; https://www.ncbi.nlm.nih.gov/, SRR28520204; https://www.ncbi.nlm.nih.gov/, SRR28520203; https://www.ncbi.nlm.nih.gov/, SRR28520202; https://www.ncbi.nlm.nih.gov/, SRR28520201; https://www.ncbi.nlm.nih.gov/, SRR28520200; https://www.ncbi.nlm.nih.gov/, SRR28520209; https://www.ncbi.nlm.nih.gov/, SRR28520208.

## Supplementary material

Supplementary Figure 1 Top 10 Gene Ontology (GO) terms of each category- Biological Process, Molecular Function and Cellular process among unigenes of **(A)** *M. persicae* adult **(B)** *L. erysimi* adult **(C)** *M. persicae* nymph **(D)** *L. erysimi* nymph. Numerals above the bars indicate the number of genes in that category in each aphid species.

Supplementary Figure 2 Number of differentially expressed unigenes in **(A)** adults and **(B)** nymphs annotated with different databases. Numerals above the bars indicate the number of genes in that category in each aphid species.

Supplementary Figure 3 Percent distribution of species showing homology with differentially regulated unigenes in **(A)** adults and **(B)** nymphs.

Supplementary Figure 4 Top 15 Gene Ontology (GO) terms of each category - Biological Process, Molecular Function and Cellular Process among differentially expressed unigenes between *M. persicae* and *L. erysimi* **(A)** upregulated and **(B)** downregulated unigenes in adults and **(C)** upregulated and **(D)** downregulated unigenes in nymphs. Red arrow in **(A)** indicates the category of upregulated oxidoreductases. Numerals above the bars indicate the number of genes in that category in each aphid species.

Supplementary Figure 5 Top 15 differentially expressed enzyme categories between *M. persicae* and *L. erysimi* in **(A)** adults and **(B)** nymphs. Red arrows indicate important enzyme categories associated with detoxification and feeding. Numerals beside the bars indicate the number of genes in that category in each aphid species.

Supplementary Figure 6 Top 15 KEGG pathways associated with differentially expressed unigenes in **(A)** adults and **(B)** nymphs of *M. persicae* and *L. erysimi*. Numerals beside the bars indicate the number of genes in that category in each aphid species.

Supplementary Figure 7 Number of differentially regulated unigenes in different categories of digestive enzymes in *M. persicae* relative to *L. erysimi* at **(A)** adult **(B)** nymph stages. Numerals above the bars indicate the number of genes in that category in each aphid species.
