# Supplementary figures and images for: Comparative transcriptomics of a generalist aphid, Myzus persicae and a specialist aphid, Lipaphis erysimi reveals molecular signatures associated with diversity of their feeding behaviour and other attributes
Source: Front Plant Sci. 2024 Dec 2;15:1415628. doi: 10.3389/fpls.2024.1415628 (PMC11648428; doi:10.3389/fpls.2024.1415628)

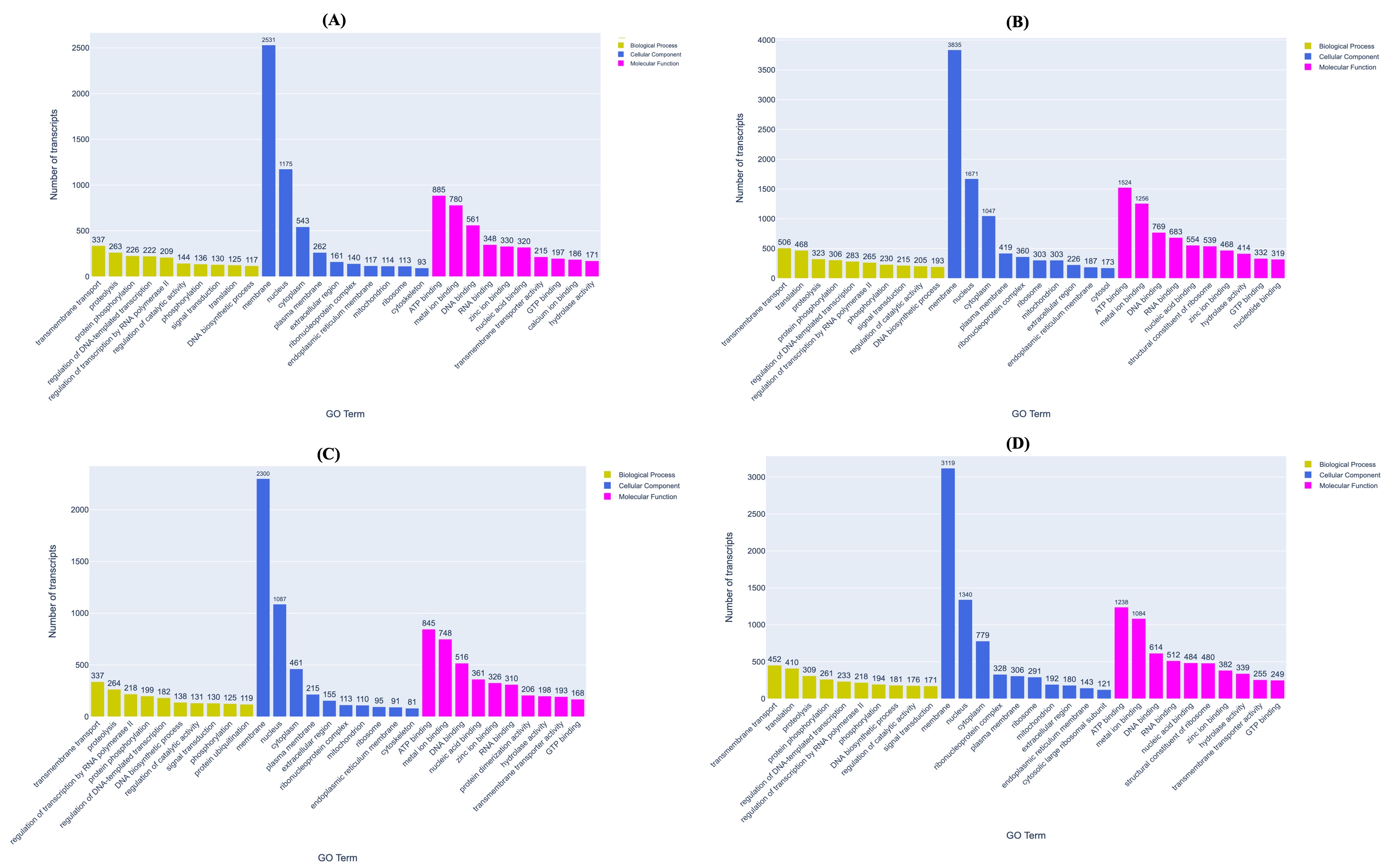

Supplement: Supplementary Figure 1 — Top 10 Gene Ontology (GO) terms of each category- Biological Process, Molecular Function and Cellular process among unigenes of (A) M. persicae adult (B) L. erysimi adult (C) M. persicae nymph (D) L. erysimi nymph. Numerals above the bars indicate the number of genes in that category in each aphid species. [file Image1.jpeg]

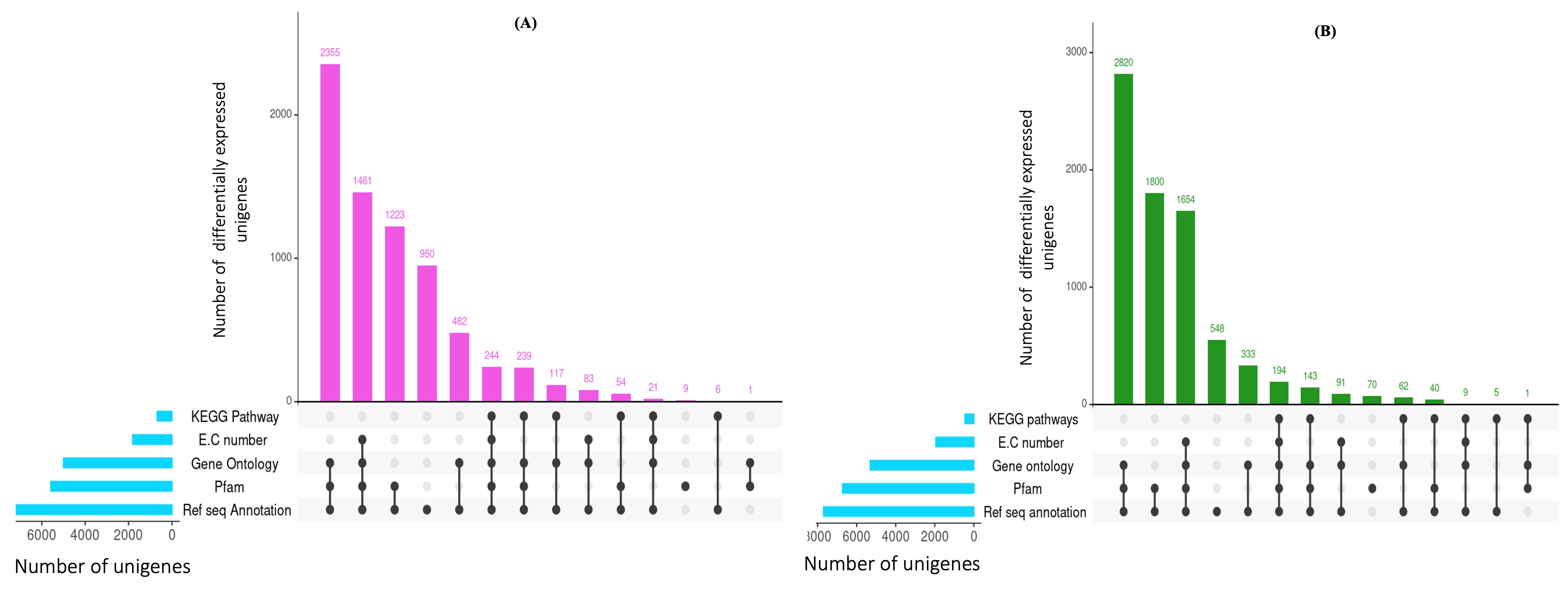

Supplement: Supplementary Figure 2 — Number of differentially expressed unigenes in (A) adults and (B) nymphs annotated with different databases. Numerals above the bars indicate the number of genes in that category in each aphid species. [file Image2.jpeg]

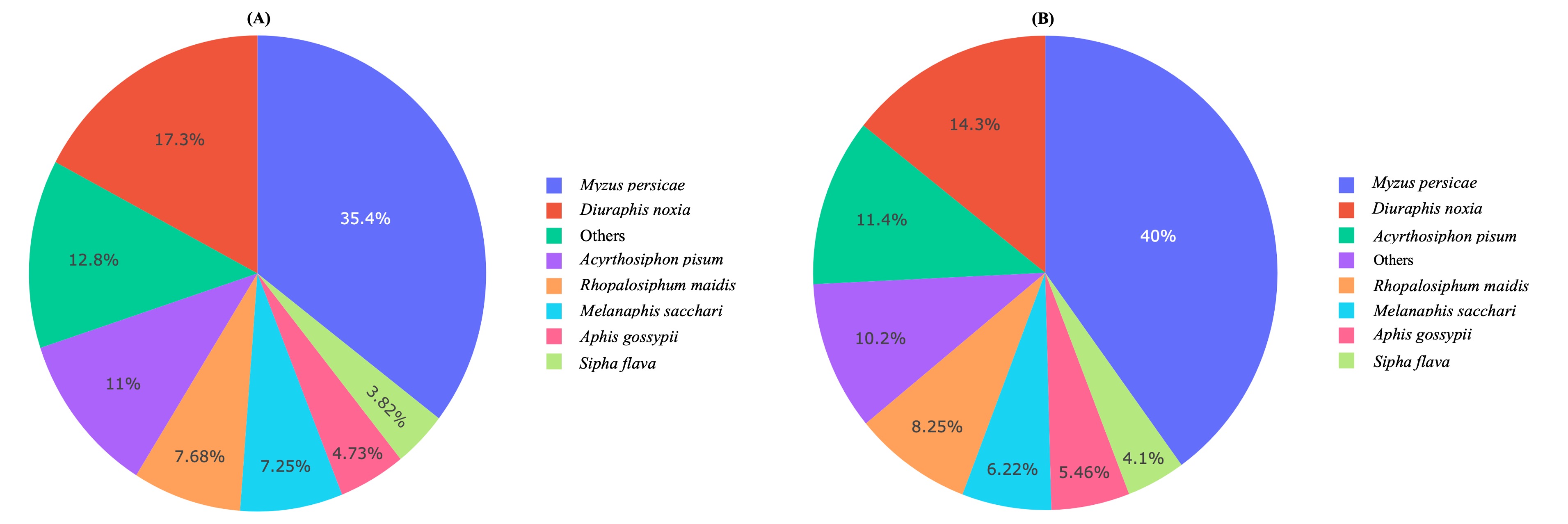

Supplement: Supplementary Figure 3 — Percent distribution of species showing homology with differentially regulated unigenes in (A) adults and (B) nymphs. [file Image3.jpeg]

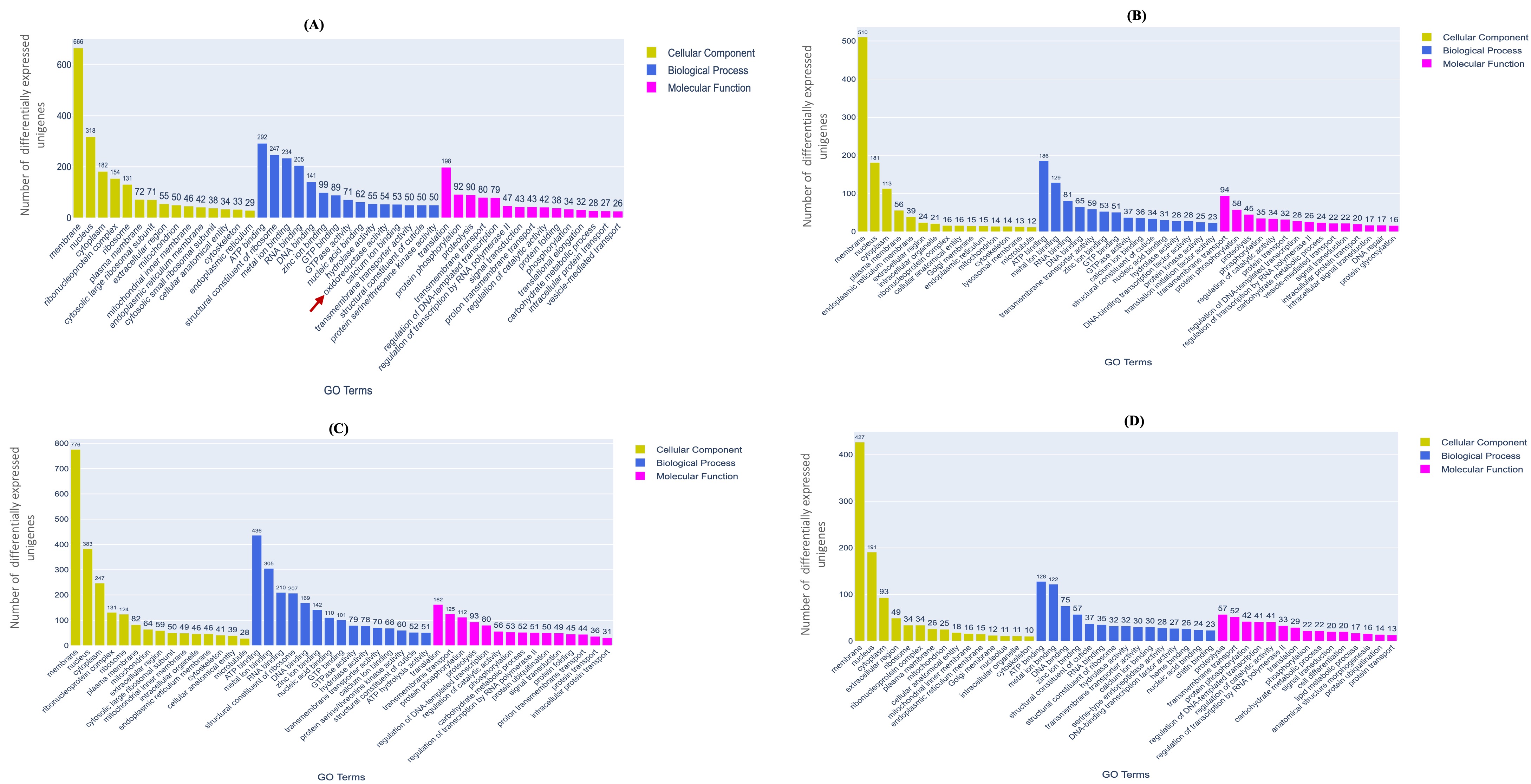

Supplement: Supplementary Figure 4 — Top 15 Gene Ontology (GO) terms of each category - Biological Process, Molecular Function and Cellular Process among differentially expressed unigenes between M. persicae and L. erysimi (A) upregulated and (B) downregulated unigenes in adults and (C) upregulated and (D) downregulated unigenes in nymphs. Red arrow in (A) indicates the category of upregulated oxidoreductases. Numerals above the bars indicate the number of genes in that category in each aphid species. [file Image4.jpeg]

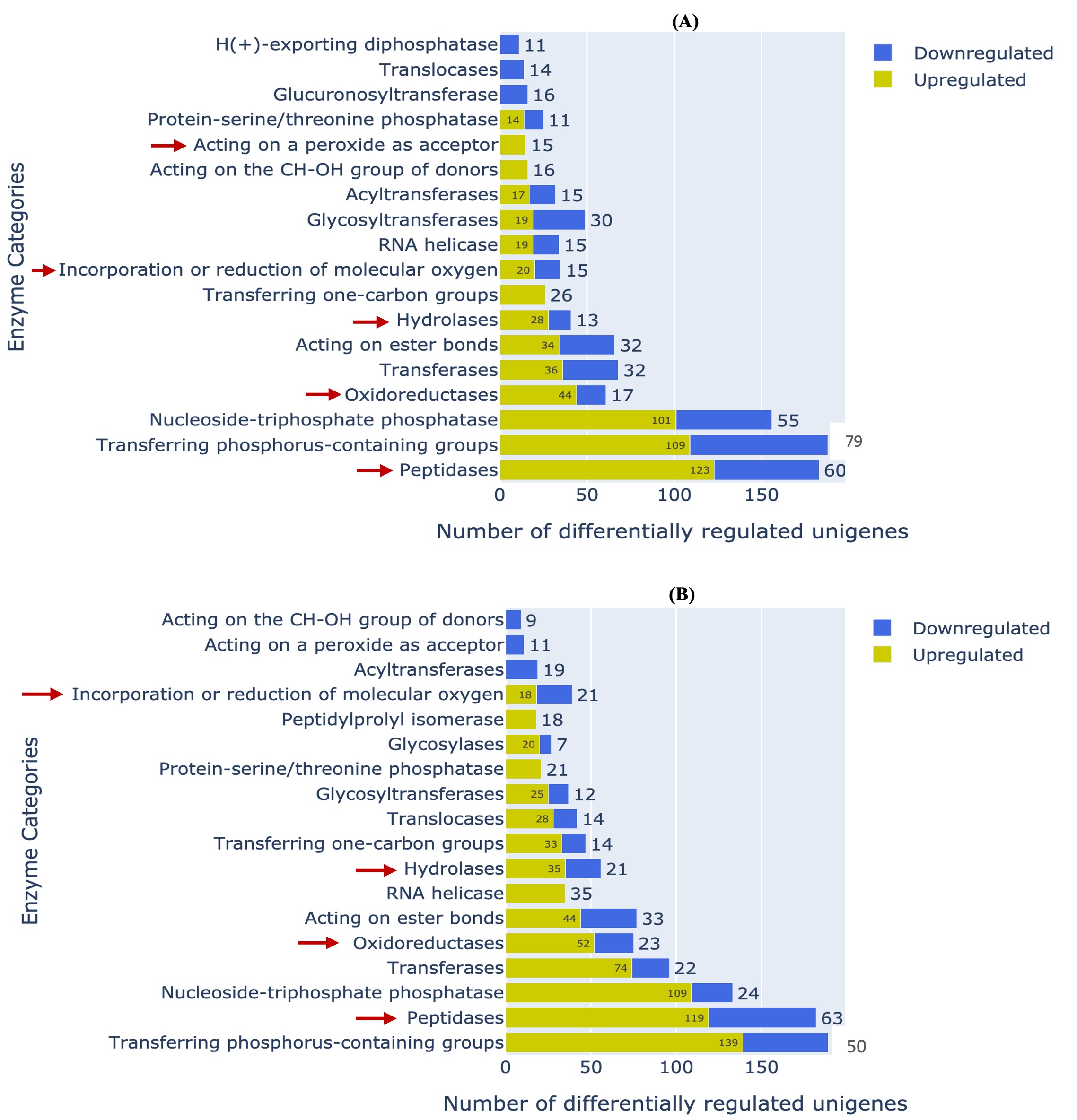

Supplement: Supplementary Figure 5 — Top 15 differentially expressed enzyme categories between M. persicae and L. erysimi in (A) adults and (B) nymphs. Red arrows indicate important enzyme categories associated with detoxification and feeding. Numerals beside the bars indicate the number of genes in that category in each aphid species. [file Image5.jpeg]

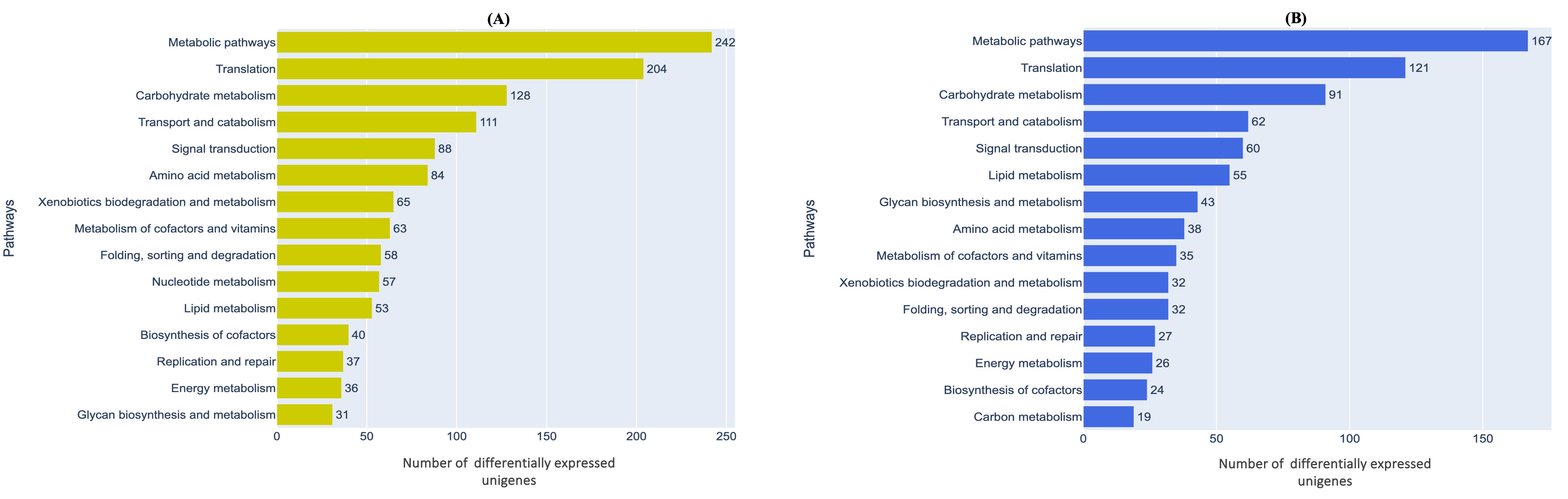

Supplement: Supplementary Figure 6 — Top 15 KEGG pathways associated with differentially expressed unigenes in (A) adults and (B) nymphs of M. persicae and L. erysimi. Numerals beside the bars indicate the number of genes in that category in each aphid species. [file Image6.jpeg]

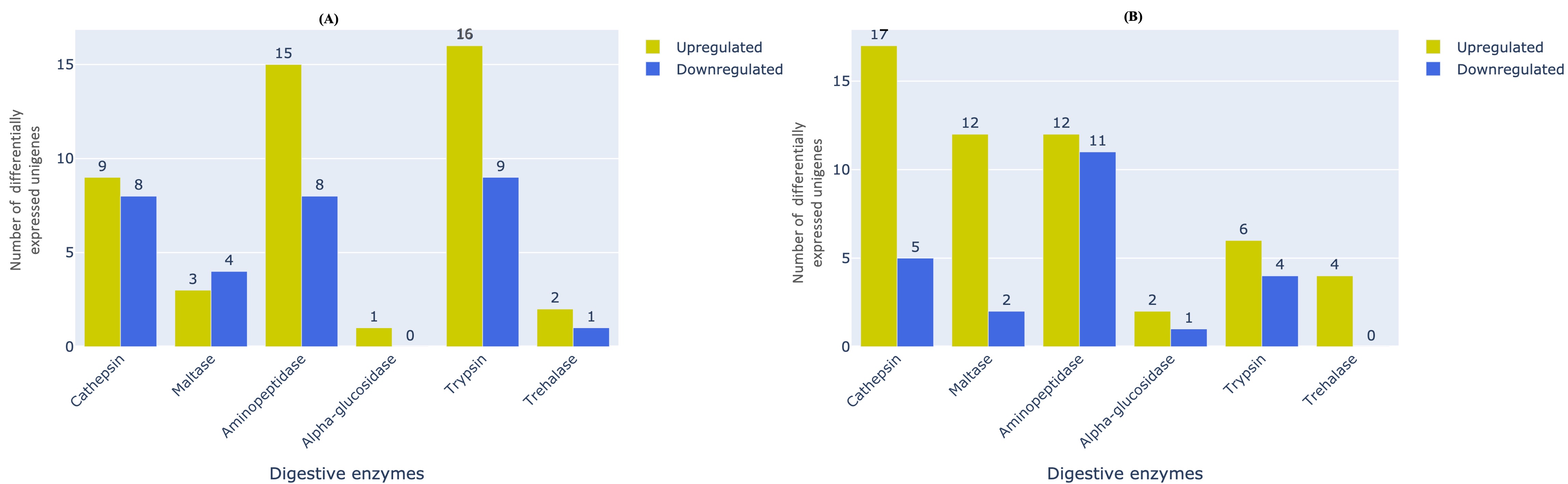

Supplement: Supplementary Figure 7 — Number of differentially regulated unigenes in different categories of digestive enzymes in M. persicae relative to L. erysimi at (A) adult (B) nymph stages. Numerals above the bars indicate the number of genes in that category in each aphid species. [file Image7.jpeg]
