# Supplementary material for: Comparative transcriptomics of a generalist aphid, Myzus persicae and a specialist aphid, Lipaphis erysimi reveals molecular signatures associated with diversity of their feeding behaviour and other attributes
Source: Front Plant Sci. 2024 Dec 2;15:1415628. doi: 10.3389/fpls.2024.1415628 (PMC11648428; doi:10.3389/fpls.2024.1415628)
Supplement: Supplementary file 11 [file Table1.docx]

Supplementary Table 1. Total number of unigenes annotated from publicly available databases

| Database | Number of annotated unigenes in *M. persicae* adult | Number of annotated unigenes in *M. persicae* nymph | Number of annotated unigenes in *L. erysimi* adult | Number of annotated unigenes in *L. erysimi* nymph |
| --- | --- | --- | --- | --- |
| Nr-Aphidbase | 15646 | 15030 | 23301 | 33656 |
| GO | 9741 | 9017 | 15535 | 12633 |
| KEGG | 98 | 102 | 3433 | 2456 |
| Pfam | 10064 | 11035 | 13627 | 13891 |
| E.C number | 3354 | 3080 | 5278 | 4122 |

Supplementary Table 2. Number of un-annotated differentially expressed unigenes between *M. persicae* and *L. erysimi* in different ranges of log2 fold changes at adult and nymphal stage.

| Ranges of fold changes (fc) | Number of un-annotated DEGs (Adult) | Number of un-annotated DEGs (Nymph) |
| --- | --- | --- |
| 2 to 8 fc | 309 | 339 |
| 8 to12 fc | 94 | 88 |
| 12 to17 fc | 53 | 150 |
| >17 fc | 62 | 26 |
| -2 to -5 fc | 49 | 107 |
